# Thermal acclimation of methanotrophs from the genus *Methylobacter*

**DOI:** 10.1038/s41396-023-01363-7

**Published:** 2023-01-18

**Authors:** Alexander T. Tveit, Andrea Söllinger, Edda Marie Rainer, Alena Didriksen, Anne Grethe Hestnes, Liabo Motleleng, Hans-Jörg Hellinger, Thomas Rattei, Mette M. Svenning

**Affiliations:** 1 Department of Arctic and Marine Biology, UiT The Arctic University of Norway, Tromsø, Norway; 2 University of Vienna, Centre for Microbiology and Environmental Systems Science, Vienna, Austria; 3 University of Vienna, Doctoral School in Microbiology and Environmental Science, Vienna, Austria

**Keywords:** Soil microbiology, Bacterial physiology, Environmental sciences

## Abstract

Methanotrophs oxidize most of the methane (CH_4_) produced in natural and anthropogenic ecosystems. Often living close to soil surfaces, these microorganisms must frequently adjust to temperature change. While many environmental studies have addressed temperature effects on CH_4_ oxidation and methanotrophic communities, there is little knowledge about the physiological adjustments that underlie these effects. We have studied thermal acclimation in *Methylobacter*, a widespread, abundant, and environmentally important methanotrophic genus. Comparisons of growth and CH_4_ oxidation kinetics at different temperatures in three members of the genus demonstrate that temperature has a strong influence on how much CH_4_ is consumed to support growth at different CH_4_ concentrations. However, the temperature effect varies considerably between species, suggesting that how a methanotrophic community is composed influences the temperature effect on CH_4_ uptake. To understand thermal acclimation mechanisms widely we carried out a transcriptomics experiment with *Methylobacter tundripaludum* SV96^T^. We observed, at different temperatures, how varying abundances of transcripts for glycogen and protein biosynthesis relate to cellular glycogen and ribosome concentrations. Our data also demonstrated transcriptional adjustment of CH_4_ oxidation, oxidative phosphorylation, membrane fatty acid saturation, cell wall composition, and exopolysaccharides between temperatures. In addition, we observed differences in *M. tundripaludum* SV96^T^ cell sizes at different temperatures. We conclude that thermal acclimation in *Methylobacter* results from transcriptional adjustment of central metabolism, protein biosynthesis, cell walls and storage. Acclimation leads to large shifts in CH_4_ consumption and growth efficiency, but with major differences between species. Thus, our study demonstrates that physiological adjustments to temperature change can substantially influence environmental CH_4_ uptake rates and that consideration of methanotroph physiology might be vital for accurate predictions of warming effects on CH_4_ emissions.

## Introduction

The only known biological CH_4_ sink are methanotrophs that utilize CH_4_ as an energy and carbon source [1, 2]. Aerobic methanotrophs belong to Gammaproteobacteria, Alphaproteobacteria, and Verrucomicrobia. These bacteria are found in soils, wetlands, water, rice paddies, landfills, sewage, and sediments, consuming subsurface biogenic or thermogenic CH_4_ or harvesting the gas directly from air [3]. Methanotrophs oxidize CH_4_ using a cytoplasmic soluble methane monooxygenase or a membrane-bound particulate methane monooxygenase (sMMO or pMMO), and fixate carbon via the ribulose monophosphate pathway, serine pathway or the Calvin-Benson-Bassham Cycle [3].

Members of the gammaproteobacterial genus *Methylobacter* have been identified as the most abundant and active methanotrophs in many ecosystems including peat soils, tundra, ponds, lakes, sub-glacial sediments, lake sediments, rice paddies, and landfills [4–11]. These environments are among the biggest contributors to global methane (CH_4_) emissions, responsible for 431 Tg CH_4_ yr^-1^ (bottom up estimated global mean for years 1978–2019) [12]. High-emitting ecosystems often have high dissolved CH_4_ concentrations (>100 µM) [13–16], meaning that *Methylobacter* species are often exposed to, and presumably adapted to, CH_4_ uptake saturation.

Soils experience a mix of stable temperatures and large temperature fluctuations driven by diurnal cycles, weather changes and season, and depending on soil type, depth, latitude, and altitude [17–20]. Highly variable temperature effects on soil CH_4_ oxidation rates have been observed, ranging from, for example, strong temperature responses in landfills [21] and marine sediments [22] to variable responses in permafrost soils [23] and minor effects under atmospheric CH_4_ concentrations in forest soils [24] or high CH_4_ concentrations in peat [25]. Temperature responses in methanotrophs have been suggested to depend on the soil type and CH_4_ concentration [26]. Also, CH_4_ oxidation has been observed to be more temperature sensitive than CH_4_ production in active layer tundra soils, but the underlying cellular mechanisms of this temperature sensitivity were not investigated [23]. Despite the apparent importance of microbial thermal acclimation (also known as thermal acclimatization and thermal adaptation [27, 28]), we do not yet have a clear understanding of how methanotrophs adjust physiologically to temperature changes and how this relates to substrate turnover and growth.

Temperature responses in bacteria occur at many levels, for example, genetic changes that influence the amino acid composition of enzymes [29], horizontal gene transfer that influences cell structure, metabolic potential, or enzyme kinetics [30], modifications of membrane fatty acid composition [31], and changes in gene expression patterns or regulatory networks [32]. For example, in response to short-term cold shock, some bacteria adapt DNA curvature and favor translation of cold-response transcripts [33]. Bacterial cells have also been shown to grow to larger sizes and sustain larger intracellular pools of ATP to compensate for kinetic limitations at low temperatures [34, 35]. Some studies suggest that accumulation of carbon and energy storage polymers can have a role in thermal acclimation of microorganisms, as seen by, for example, the transcriptional upregulation of polyhydroxyalkanoate (PHA) storage in anoxic peat at low temperature (<10 °C) [36], PHA accumulation at high temperature (30–37 °C) in methanotrophic enrichments dominated by *Methylocystis* [37], and glycogen storage as a survival mechanism at low temperature in *E. coli* [38].

Central to the cellular response of bacteria to altered conditions, including temperatures, is the genetic information processing machinery [39], including the transcription of DNA into RNA and the translation of RNA into proteins. Ribosomal tuning was shown through studies on *E. coli* to be a major cellular tool to optimize growth rates and physiological states in response to changes in their external conditions, including temperature (e.g., [40, 41]). Recently, studies of soil microbial responses to long-term warming have revealed that the higher growth rates at higher temperatures were accompanied by a reduction in the number of ribosomes [42], confirming that adjustment of the protein biosynthesis machinery is an important thermal acclimation mechanism in nature. However, so far, our understanding of thermal acclimation via ribosomal regulation is mostly limited to studies on the model organism *E. coli*.

Due to a considerable CH_4_ uptake, including a consumption of up to 90% of produced CH_4_ in wetlands [43] and atmospheric CH_4_ uptake in soils [44], methanotrophs constitute one of the most important CH_4_-sinks on Earth [45]. As these microorganisms are frequently exposed to temperature change in soil surface layers, their means of physiological adjustment may significantly influence global CH_4_ cycling. Based on the frequent reports of their detection [3], members of the genus *Methylobacter* may be a particularly important contributor to the biological CH_4_ sink. However, our knowledge of temperature effects on methanotroph physiology is very limited.

Here, we have investigated how *Methylobacter* acclimate to different temperatures and CH_4_ concentrations in comparative growth and CH_4_ oxidation kinetics experiments with strains from three *Methylobacter* species. Furthermore, to learn which cellular machineries are adjusted for thermal acclimation, we performed a series of temperature experiments including transcriptomics and measurement of cell sizes and cell contents with one of these strains, *Methylobacter tundripaludum* SV96^T^.

## Results and discussion

### Environmental distribution of *Methylobacter* and temperature effects on growth and CH_4_ oxidation kinetics in three *Methylobacter* strains

By screening the data available in the Earth Microbiome Project (EMP) [46], we observed a widespread distribution of *Methylobacter*-like 16S rRNA gene sequences (Fig. S1), covering 17% of all 23,813 screened EMP samples (Supplementary dataset (SD) A: Table A1) and including 256 out of 1355 unique geographic locations (SD A: Table A2). *Methylobacter-like sequences* were most frequently observed in freshwater (64% of all freshwater samples in the database) (SD A: Table A3) and were also most abundant in freshwater (SD A: Table A1). In line with this, members of *Methylobacter* are often observed in environmental studies [3] and the type strain species *M. tundripaludum* (SV96^T^) [47] is common in wetlands, lakes, and other high-CH_4_ environments [4–7, 48]. The high abundance and global distribution *Methylobacter* in freshwater environments, suggests a globally important role in CH_4_ cycling as these are hotspots for CH_4_ production, contributing ~42% of total anthropogenic and natural CH_4_ emissions [49].

To study how thermal acclimation affects growth and CH_4_ oxidation in these environmentally important microorganisms we compared growth kinetics and CH_4_ oxidation kinetics of three different *Methylobacter* strains, *Methylobacter tundripaludum* SV96^T^, *Methylobacter* sp. G7, and *M. luteus* ACM 3304 ^T^ (see Fig. S2 for experimental setup).

**Fig. 1:**
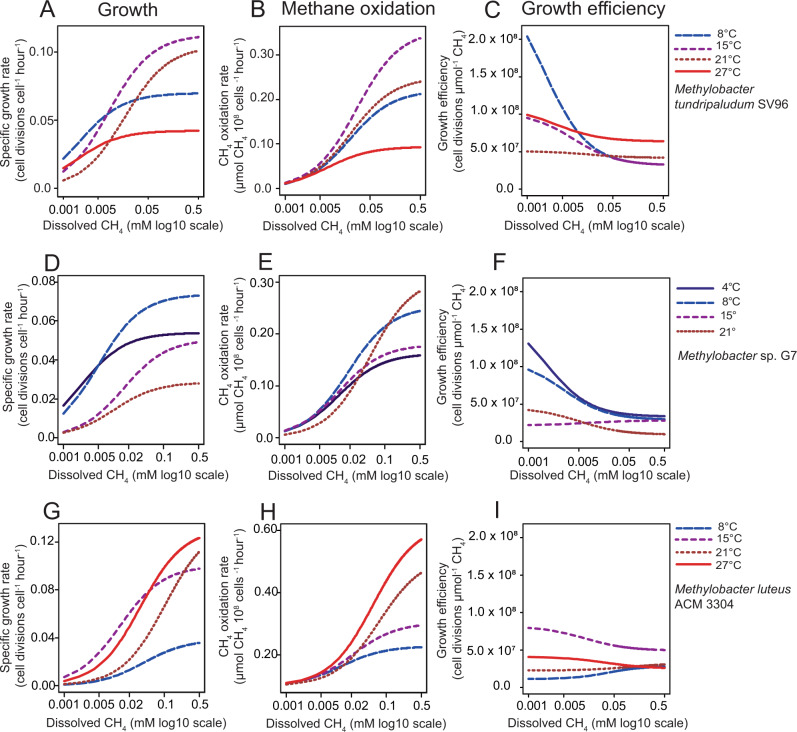
Growth kinetics, CH_4_ oxidation kinetics and growth efficiency of Methylobacter strains at different temperatures. Growth kinetics, CH_4_ oxidation kinetics and growth efficiency at different temperatures for *M. tundripaludum* SV96^T^ (**A**, **B**, **C**), *Methylobacter* sp. G7 (**D**, **E**, **F**) and *Methylobacter luteus* ACM 3304 ^T^ (**G**, **H**, **I**). **A** Michaelis-Menten kinetics models of specific growth rates (cell divisions per cell per hour) at different dissolved CH_4_ concentrations and different temperatures (8, 15, 21, and 27 °C) for *M. tundripaludum* SV96^T^. The *x*-axis is on a log10 scale to better resolve the differences between temperatures in the low and high end of concentrations. Data, and individual parameters and statistics for each model is found in Fig. S4 and SD B, Table B1. **B** Michaelis-Menten kinetics models of CH_4_ oxidation rates per cell for different dissolved CH_4_ concentrations and temperatures (8, 15, 21, and 27 °C). Data, and individual parameters and statistics for each model is found in Fig. S4 and SD B, Table B2. Standard errors and *p* values for the coefficient estimates, and residual standard errors of the growth and CH_4_ oxidation models were estimated using non-linear regression, applying a Michaelis-Menten function (see the Materials and Methods section “Statistics for physiological measurements”). **C** Cell divisions per µmol CH_4_ oxidized at different dissolved CH_4_ concentrations and temperatures. Growth efficiency estimates were calculated from the predicted rates in (**A**) and (**B**) by dividing specific growth rate predictions by CH_4_ oxidation rate predictions to obtain cell divisions per µmol CH_4_ oxidized. Four-parameter logistics curves (dose-response model) were fitted to the resulting quotients. The *p* values for the four parameters (min, max, inflection and hill) of the dose-response models for different temperatures are shown in Fig. S4 and SD B, Table B3. **D**–**F** Same as for (**A**–**C**), except *Methylobacter* sp. G7 was incubated at 4, 8, 15, and 21 °C. Data and individual models are found in Fig. S5 and SD B, Tables B5, B6, B7. **G**–**I** Same as for (**A**–**C**). *Methylobacter luteus* ACM 3304 ^T^ was incubated at the same temperatures at *M. tundripaludum* SV96^T^. Data and individual models are found in Fig. S6 and SD B, Tables B8, B9, B10. Each of the nine figures is based on 84 incubations with 4–5 timepoint measurements per incubation.

*Methylobacter tundripaludum* SV96^T^ originates from Arctic peat and its temperature range for growth suggests psychrotolerance with a growth optimum between 15 and 25 °C [47]. Phylogeny of its *pmoA* gene (common marker gene encoding the beta subunit of pMMO) places this strain in a different clade than most of the characterized *Methylobacter* species, including *Methylobacter luteus* and *Methylobacter whittebury* (Fig. S3) as previously also shown using phylogenomics [50]. In experiments with *M. tundripaludum* SV96^T^, we compared growth and CH_4_ oxidation Michaels-Menten kinetics between 8, 15, 21, and 27 °C, across seven dissolved CH_4_ concentrations ranging from 0.003 to 0.25 mM (*n* = 84 incubations with ~4 timepoint measurements per incubation). At CH_4_-uptake saturation (V_max(app)_: “app” indicating cellular V_max_), specific growth was fastest at 15 °C, followed by 21, 8, and 27 °C (Fig. 1A, Fig. S4, SD B: Table B1). At CH_4_ concentrations below ~0.005 µM, the specific growth rate at 8 °C was higher than at the other temperatures (Fig. 1A, Fig. S4). CH_4_ oxidation rates also changed with both temperature and CH_4_ concentration (Fig. 1B, SD B: Table B2). At CH_4_-uptake saturation, highest cellular CH_4_ consumption rates (V_max(app)_) were detected at 15 °C, followed by 21, 8, and 27 °C (Fig. 1B and Fig. S4, SD B: Table B2). At lower CH_4_ concentrations (below 0.005 µM), we observed only minor differences in CH_4_ consumption between temperatures. Consequently, the growth efficiency (number of cell divisions per CH_4_ molecules oxidized, modelled with four-parameter logistics curves) at CH_4_ saturation was much lower at 8 and 15 °C than at 21 and 27 °C (Fig. 1C, SD B: Table B3). However, growth efficiency increased with decreasing CH_4_ concentrations at all temperatures, with the largest increase at 8 °C, followed by 15, 27, and 21 °C, respectively. This observation, that at high CH_4_ concentrations the highest growth efficiency was found at high temperatures (21 and 27 °C), while at low CH_4_ concentrations the highest growth efficiency was found at low temperatures (8 and 15 °C), implies large shifts in cellular resource allocation and therefore CH_4_ consumption due to changes in temperature and CH_4_ concentration. This also disagrees with a proposed model in which fast and slow growth are suggested to be inefficient while the highest efficiency is obtained at intermediate growth rates [51].

*Methylobacter* sp. G7 is closely related to *M. tundripaludum* SV96^T^, *pmoA* gene phylogeny placing these strains within the same clade (Fig. S3). It originates from a biofilm inside the coalmine G7, close to Longyearbyen, Svalbard (78°13′00″N 15°38′00″E). *Methylobacter* sp. G7 has not been published as a novel species to date, but its gene identities to *M. tundripaludum* SV96^T^ (94.4% for *pmoA* and 98.9% for the 16 S rRNA gene) and different phospholipid fatty acid (PLFA) profile compared to *M. tundripaludum* SV96^T^ (SD B: Table B4) suggest that it may represent a novel *Methylobacter* species. The experiments with *Methylobacter* sp. G7 were set up the same way as for *M. tundripaludum* SV96^T^ (*n* = 84). However, this strain did not grow at 27 °C, and therefore these experiments were carried out at 4, 8, 15, and 21 °C. The highest growth rate for *Methylobacter* sp. G7 under CH_4_ saturation was at 8 °C, followed by 4, 15, and 21 °C (Fig. 1D, Fig. S5, SD B: Table B5). The highest CH_4_ oxidation rates, under CH_4_ saturation, were at 21 and 8 °C, followed by 15 and 4 °C (Fig. 1E, Fig. S5, SD B: Table B6). This indicates the highest growth efficiency at 4 °C, followed by 8, 15, and 21 °C, when CH_4_ concentrations are saturated (Fig. 1F, Fig. S5, SD B: Table B7), opposite to that of *M. tundripaludum* SV96^T^ (Fig. 1C). However, like *M. tundripaludum* SV96^T^, the growth efficiency of *Methylobacter* sp. G7 increased substantially with decreasing CH_4_ concentrations.

*Methylobacter luteus* is a mesophilic species that clusters separately from the two other strains (Fig. S3) and is found in, for example, freshwater, marine sediments, sewage, and landfills [48, 52]. *M. luteus* ACM 3304 ^T^ has a different PLFA profile than the two other strains (Supplementary dataset B, Table B4). The experiments (*n* = 84) with *M. luteus* ACM 3304 ^T^ (originally isolated from sewage [3]) demonstrated the highest cell division and CH_4_ oxidation rates under CH_4_ saturation at 27 °C, followed by 21, 15, and 8 °C (Fig. 1G and H, Fig. S6, SD B: Tables B8 and B9). The highest growth efficiency under CH_4_ saturation was observed at 15 °C (Fig. 1I, Fig. S6, SD B: Table B10), followed by 21, 8, and 27 °C. We observed only small changes in growth efficiency with changing CH_4_ concentration in *M. luteus* ACM 3304 ^T^, and apart from the large increase in growth efficiency at 15 °C, we saw little change with temperature, confirming that the thermal acclimation strategy of *M. luteus* ACM 3304 ^T^ is very different from the two other strains. However, a large influence of temperature on CH_4_ consumption per cell was common to all three strains (Fig. 1B, E, H).

By comparing the growth efficiencies of the three *Methylobacter* strains we can demonstrate how thermal acclimation can affect CH_4_ uptake rates. For example, we see that substantial additional quantities of CH_4_ are consumed by *M. tundripaludum* SV96^T^ at 15 and 8 °C to support growth at higher CH_4_ concentrations, relative to 21 and 27 °C. The same is seen at 21 °C relative to the other temperatures for *Methylobacter* sp. G7 (Fig. 1B and E). Maximizing growth rates can be physiologically inefficient, for example in cases where more proteins are required to speed up the rates [40], but this strategy can be beneficial when sufficient resources are available. On the other hand, *M. tundripaludum* SV96^T^ and *Methylobacter* sp. G7 have both evolved acclimation strategies where the most efficient growth occurs at low temperatures and low CH_4_ concentrations (Fig. 1C and F). Efficient growth means lower CH_4_ oxidation rates, and thus our results suggest that transitions from inefficient to efficient growth due to temperature changes in nature can lead to lower CH_4_ oxidation rates, or vice versa. For *M. luteus* ACM 3304 ^T^, the effect of temperature on CH_4_ consumption is especially evident in the much more efficient growth at 15 °C relative to the other temperatures. This implies that a drop in temperature from 25 to 15 °C would reduce the size of a *M. luteus* ACM 3304^T^-dominated CH_4_ sink considerably, on a per cell basis, not because the strain cannot function at the temperature, but because it is growing more efficiently. This further indicates that the competitiveness of a strain does not necessarily correlate with its CH_4_ consumption. Thus, while CH_4_ production rates are often expected to increase with increasing temperatures [53], our data demonstrate that CH_4_ oxidation rates might not always correlate, for physiological reasons, possibly explaining some of the divergent observations of temperature effects on CH_4_ oxidation in environmental studies (e.g., [23, 25]).

### Cell sizes and RNA and DNA content of *M. tundripaludum* SV96^T^

**Fig. 2:**
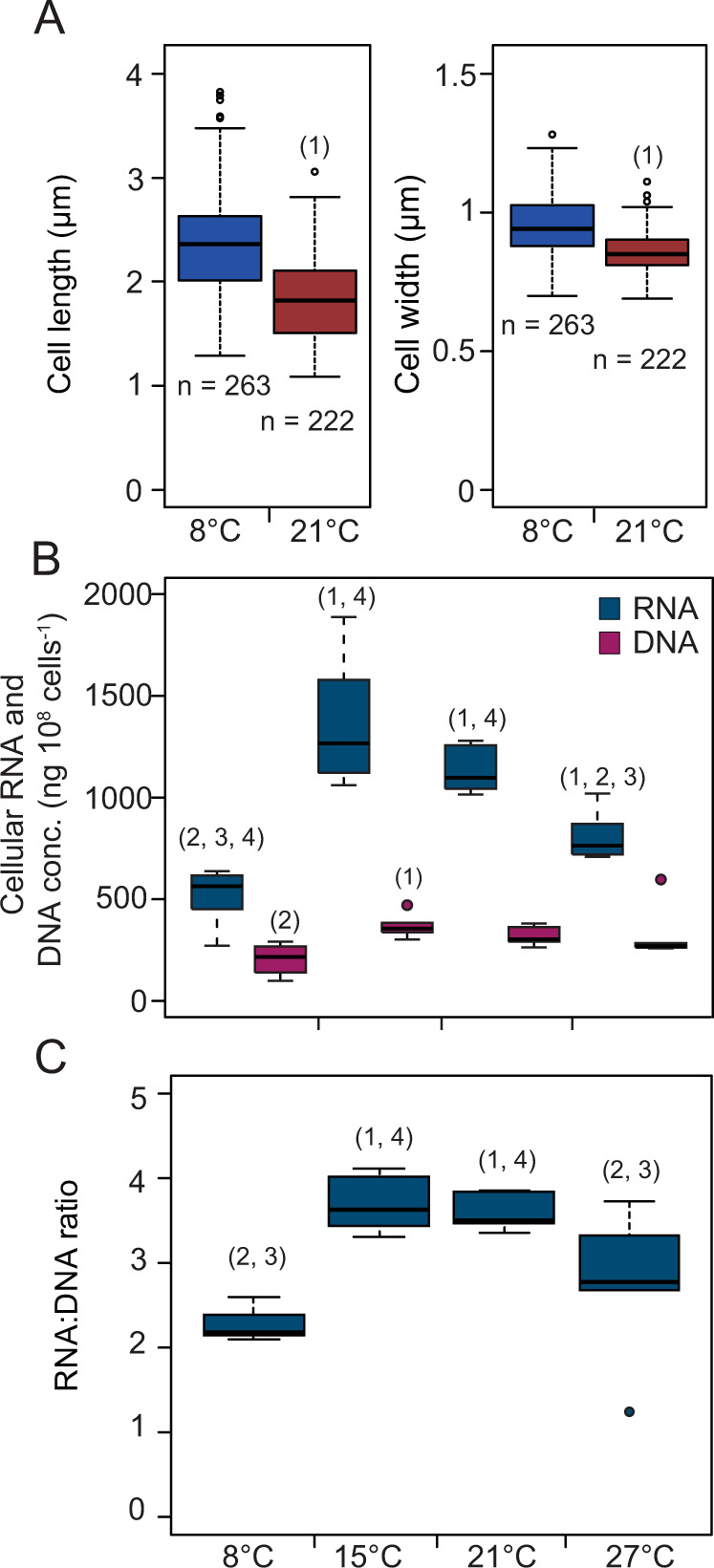
Cell sizes and RNA (blue) and DNA (purple) content of *M. tundripaludum* SV96^T^ at different temperatures. In (**A**), boxplots show the differences in cell length (left) and cell width (right) between 8 and 20 °C. In (**B**), boxplots show the concentrations of RNA and DNA in *M. tundripaludum* SV96^T^ cells. In (**C**), boxplots show the RNA:DNA ratios. Differences between means were evaluated using unpaired one-tailed *t*-tests. Significant differences (*p* < 0.05, multiple testing correction using the approach of Benjamini-Hochberg) to 8 °C (1), 15 °C (2), 21 °C (3) and 27 °C (4) are indicated with numbers above the individual boxes. The corresponding data can be found in SD B: Table B12.

We directed our attention toward *M. tundripaludum* SV96^T^ for investigating in more depth the cellular mechanisms that are involved in thermal acclimation. For many decades, observations have been made of microorganisms, including algae and bacteria, that change size in response to temperature (e.g., [54, 55]). For the temperatures 8 and 20 °C, we tested whether this was also the case for *M. tundripaludum* SV96^T^. We observed slight, but significant, decreases of width (5% on average, *n* = 222–263) and length (10% on average, *n* = 222–263) with increasing temperature (Fig. 2A). However, we did not see consistent differences in optical density of the cultures despite these cell size differences, possibly due to the combination of low percentage change and technical variability in optical density measurements.

We then prepared for running a transcriptomics experiment at 8, 15, 21, and 27 °C and saturating CH_4_ concentrations (>0.1 mM dissolved CH_4_) during exponential growth. A growth experiment (Fig. S7A, SD B, Table B11) was first carried out to test whether the same temperature response could be observed under these conditions as in the growth kinetics experiment (Fig. 1A and Fig. S4). The results of the growth experiment showed no significant differences between the growth rates at 8 and 27 °C (0.035 and 0.038 cell divisions cell^−1^ h^−1^, respectively), and no significant differences between the rates at 15 and 21 °C (0.085 and 0.095 cell divisions cell^−1^ h^−1^, respectively). While these rates were lower than indicated by the model predictions from the growth kinetics experiment (Growth V_max(app)_: 0.0699 and 0.0612 cell divisions cell^−1^ h^−1^ at 8 °C and 27 °C, and 0.1128 and 0.1044 cell divisions cell^−1^ h^−1^ at 15 and 21 °C, respectively), the ratios between the rates at different temperatures were similar. This confirmed that temperature responses were consistent and thus comparable between experiments, so we proceeded to extract total nucleic acids from the cells incubated for transcriptomics. Prior to RNA purification we measured the quantities of RNA and DNA. The DNA quantities per 10^8^ cells did not significantly differ between temperatures (*p* > 0.08) while the RNA quantities were significantly different between all temperatures (*p* < 0.05), except 21 and 15 °C (*p* = 0.06), with highest RNA content at 15 and 21 °C, followed by 27 and 8 °C (Fig. 2B, SD B: Table B12). Correspondingly, the RNA to DNA ratio in *M. tundripaludum* SV96^T^ varied between temperatures, with mean of 2.3, 3.7, 3.6 and 2.8, at 8, 15, 21, and 27 °C, respectively (Fig. 2C, SD B: Table B12) and RNA:DNA ratios were significantly lower at 8 and 27 °C compared to 15 and 21 °C (*p* < 0.05). Changes in cellular total RNA reflect changes in ribosomal RNA (rRNA) as most bacterial RNA is rRNA (82–90%) [56]. Ribosomal RNA can constitute up to 20% of cell dry weight [57] and ribosomal proteins make up 20–40% of total proteins [40]. Thus, tuning of the cellular ribosome concentration is a key factor in optimizing growth rates in *E. coli* [40]. Our results suggest that *M. tundripaludum* SV96^T^ reduces its cellular rRNA concentration at the higher (27 °C) and lower (8 °C) end of the temperature range that allows growth, while maintaining similar concentrations in the optimum range (15 and 21 °C). Similarly, in *E. coli*, cellular ribosome concentrations were suggested to remain constant at temperatures around the growth optimum but decline at higher and lower temperatures as some of the resources were diverted toward other physiological processes, including stress responses, that are needed to maintain high growth rates under sub-optimal conditions [58].

### Central carbon and energy metabolisms

To identify which physiological components, other than rRNA, are adjusted for thermal acclimation in *M. tundripaludum* SV96^T^, we sequenced three mRNA enriched transcriptomes from each of the temperatures (Fig. S2A), resulting in a total of 12 mRNA libraries. The mRNA fraction ranged from 30 to 99% mRNA after rRNA depletion, leaving between 2.2 million and 15.3 million mRNA reads aligned to the *M. tundripaludum* SV96^T^ genome (SD B: Table B13).

**Fig. 3:**
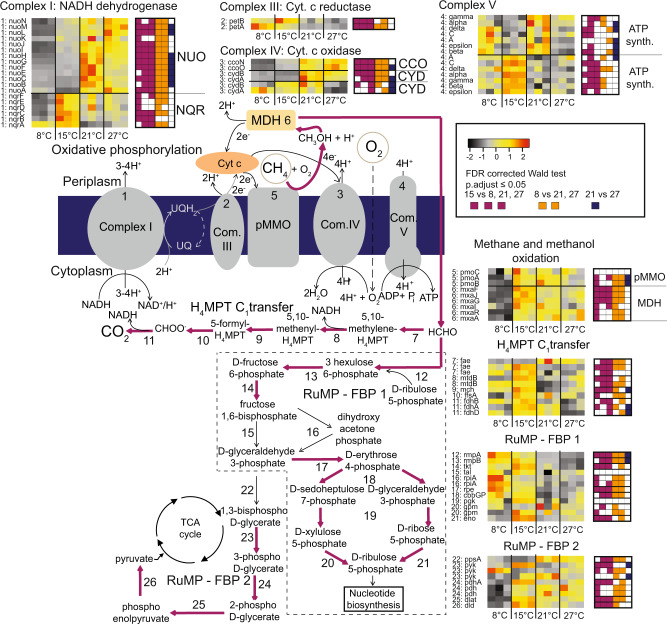
Gene expression for central carbon and energy metabolisms in *M. tundripaludum* SV96^T^. Heatmaps indicate changes in the relative abundance of transcripts in the transcriptomes at 8, 15, 21, and 27 °C. The color scale reflects *z*-score normalized relative abundances, with black being lowest relative abundance, followed by gray, yellow, orange, and red, corresponding to increasing relative abundances. Significant differences (*p* < 0.05) in transcript relative abundances are displayed in square plots to the right of each heatmap, purple indicating significant difference between 15 °C, and 8, 21 or 27 °C, respectively, when moving from left to right. Orange indicates significant differences between 8 °C and 21 and 27 °C. Dark blue indicates significant differences between 21 and 27 °C. All *p* values were estimated with a Wald test, implemented in DESeq2, and corrected for multiple testing using Benjamini Hochberg correction which adjusts for the false discovery rate. Purple arrow in metabolic pathways indicates upregulation at 15 °C, meaning that at least one of the genes encoding the enzyme responsible for catalyzing the reaction represented by this pathway step is significantly higher expressed at 15 °C than at two or three of the other temperatures. Numbers next to heatmaps refer to corresponding genes in SD C: Table C1 and corresponding metabolic step illustrated in the figure. All adjusted *p* values, normalized counts, genome IDs (a unique identifier that allows tracking of each gene to the genome annotation available in https://mage.genoscope.cns.fr/microscope), full protein names and E.C. numbers for the genes are provided in SD C: Table C1.

We observed upregulation (upregulation or increased gene expression referring to significantly increased transcript numbers) of genes encoding pMMO and methanol dehydrogenase at 15, 21, and 27 °C compared to 8 °C (Fig. 3, SD C: Table C1). The high oxidation rates (V_max(app)_) at 8 °C (Fig. S4) despite low expression of pMMO (Fig. 3) and fewer ribosomes per cell at this temperature (Fig. 2B) relative to 21 and 27 °C (Fig. S4), suggest that *M. tundripaludum* SV96^T^ may carry a low temperature-adapted pMMO. The combination of high pMMO expression (Fig. 3) and high cellular ribosome content (Fig. 2) at 15 °C corresponded to the highest cellular CH_4_ oxidation rate (V_max(app)_ = 0.36 µmol CH_4_ 10^8^ cells^−1^ h^−1^) (Fig. S4). However, at 21 °C, similarly high ribosome content and pMMO expression as at 15 °C resulted in a much lower cellular V_max(app)_ (Fig. S4), also supporting the proposition of a low temperature-adapted pMMO. Perhaps an inefficient pMMO at high temperature is one of the reasons for the overall higher pMMO gene expression at temperatures above 8 °C.

We observed upregulation at 15 °C of genes in the NADH-generating tetrahydromethanopterin (H_4_MPT) pathway, used for C_1_ transfer during formaldehyde oxidation to CO_2_ (Fig. 3). This may suggest higher rates of energy conservation. However, there was no consistent upregulation of oxidative phosphorylation at 15 °C (Fig. 3). Rather, we observed increased expression, at different temperatures, of genes for different enzyme systems with the same functional role (e.g., the NUO and NQR versions of the NADH dehydrogenase complex I). Isozymes, enzymes with different amino acid sequences that catalyze the same reaction, have previously been suggested as an important part of microbial thermal acclimation [27, 59]. However, the discrete functional roles of different functionally analogous but non-homologous enzyme systems with roles in oxidative phosphorylation are generally not well understood [60].

At 15 °C, we also observed upregulation of a large proportion of the genes leading from CH_4_ oxidation through the Ribulose monophosphate pathway (RuMP) fructose bisphosphate branch 1 (FBP 1) toward nucleotide biosynthesis (Fig. 3). Considering that nucleotides are the main building blocks for RNA, this matched the high cellular RNA content at 15 °C (Fig. 2B).

### Nucleotide biosynthesis, transcription, and translation

**Fig. 4:**
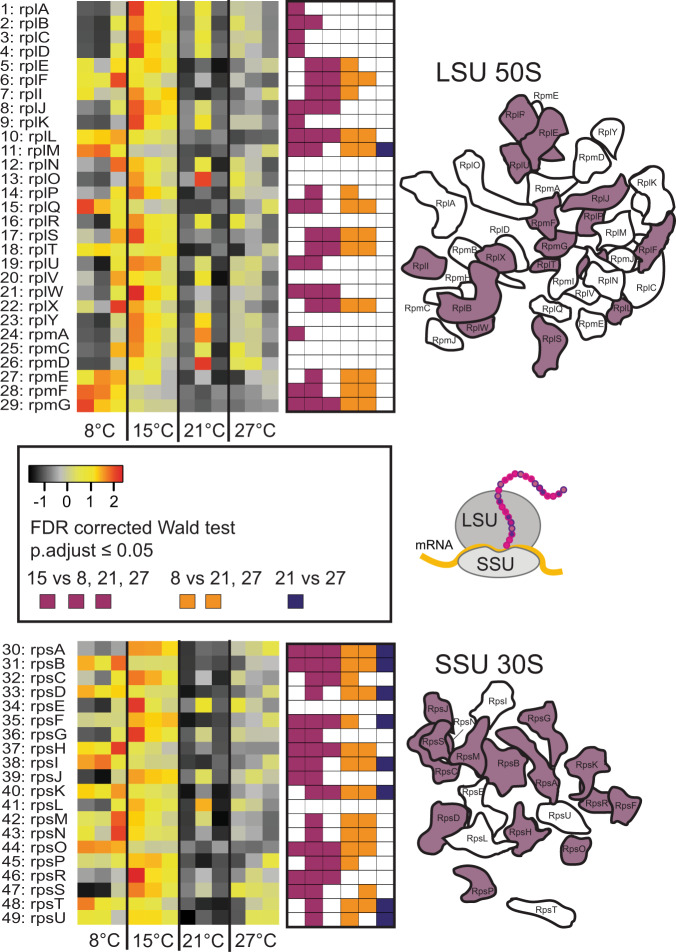
Gene expression for ribosomal proteins in *M. tundripaludum* SV96^T^. Heatmaps indicate changes in the relative abundance of transcripts in the transcriptomes at 8, 15, 21, and 27 °C. The color scale reflects *z*-score normalized relative abundances, with black being lowest relative abundance, followed by gray, yellow, orange, and red, corresponding to increasing relative abundances. Significant differences (*p* < 0.05) in transcript relative abundances are displayed in square plots to the right of each heatmap, purple indicating significant difference between 15 °C and 8, 21 and 27 °C, respectively, when moving from left to right. Orange indicates significant differences between 8 °C and 21 and 27 °C. Dark blue indicates significant differences between 21 and 27 °C. All *p* values were estimated with a Wald test, implemented in DESeq2, and corrected for multiple testing using Benjamini Hochberg correction which adjusts for the false discovery rate. Ribosomal proteins that are colored purple in the visualization of the ribosomal proteins on the right of the heatmaps indicate that the genes encoding these proteins are significantly higher expressed at 15 °C than at two or three of the other temperatures. The ribosomal protein overview is modified from [74]. All adjusted *p* values, normalized counts, genome IDs (a unique identifier that allows tracking of each gene to the genome annotation available in https://mage.genoscope.cns.fr/microscope) and full protein names are given in SD C: Table C4. Rpl and rpm: large ribosomal subunit proteins; rps: small ribosomal subunit proteins. The ribosome cartoon is partly based on previous illustrations [42]. Numbers next to heatmaps refer to corresponding genes in SD C: Table C4.

Nucleotide biosynthesis pathways receive the precursors 2-Oxo-glutarate and D-ribulose-5P from the tricarboxylic acid cycle and RuMP, respectively. We observed upregulation at 15 °C for 30 out of 46 pathway steps needed for nucleotide biosynthesis from these two precursors, matching the upregulation of RuMP toward nucleotide biosynthesis (Fig. S8, SD C: Table C2). Among the genes encoding the DNA-directed RNA polymerase, only the β subunit, *rpoB* (α; *rpoA*, β^’^; *rpoC*, β; *rpoB*) was upregulated at 15 °C (Fig. S9, SD C: Table C3). The genes for three out of four enzymes that compose the RNA degradosome; RNase E, Enolase, and Pnpase (polynucleotide phosphorylase); were also upregulated at 15 °C (Fig. S9). Only one of the genes encoding an ATP dependent RNA helicase (*rhlB*), was upregulated at 15 °C (although not relative to 8 °C), *rhlE* was not. Furthermore, genes encoding enzymes for protein folding (*groE*, *groL*, and *dnaK*) were upregulated at 15 °C (Fig. S9). These increased investments into transcription, RNA degradation, and protein folding at 15 °C matched an overall upregulation of genes for the small subunit (SSU) and large subunit (LSU) ribosomal proteins, with 15 out of 29 LSU and 14 out of 19 SSU proteins being significantly higher expressed at 15 °C (Fig. 4, SD C: Table C4). This was further supported by the upregulation of genes for many individual amino acid biosynthesis pathway steps (43 out of 79) at 15 °C relative to the other temperatures (Fig. S10, SD C: Table C5). These findings are in accordance with previous observations of transcriptional adjustment of genes for translation, amino acid biosynthesis, nucleotide metabolism, and protein metabolism in *E. coli* exposed to changing temperatures [61, 62]. The upregulation of these genes in *M. tundripaludum* SV96^T^ was observed exclusively at 15 °C (Figs. 4, S8, S9, S10), while similar cellular rRNA concentrations were found at 15 and 21 °C (Fig. 2). Thus, it seems that to maintain a similar cellular rRNA concentration and similar growth rate at 15 and 21 °C, the cell must tune its gene expression differently. In this context, it is important to note that the overall upregulation of protein biosynthesis machinery genes at 15 °C also does not imply that we find the highest protein synthesis rates at 15 °C. The reason is that all synthesis rates, including the synthesis of ribosomes and proteins, are directly influenced by temperature [58]. However, it does imply higher protein biosynthesis rates at 15 °C, relative to the rates that would have been obtained at 15 °C without upregulation. Such catalytic compensation comes at a cost. Perhaps this is one of the reasons for the lower growth efficiency under CH_4_ saturation at 15 °C, relative to 21 °C (Figs. 1 and S4). Maybe the similar cellular rRNA concentrations and growth rates at 15 and 21 °C was only possible due to the increased cellular CH_4_ consumption and larger investment into protein biosynthesis at 15 °C. Catalytic compensation at low temperature was also previously observed, for example, in the upregulation of ATP synthesis and CO_2_ fixation via RuBisCO in bacteria and plants, respectively [34, 63], and suggested as part of the bacterial response to soil warming [42].

### Glycogen storage

**Fig. 5:**
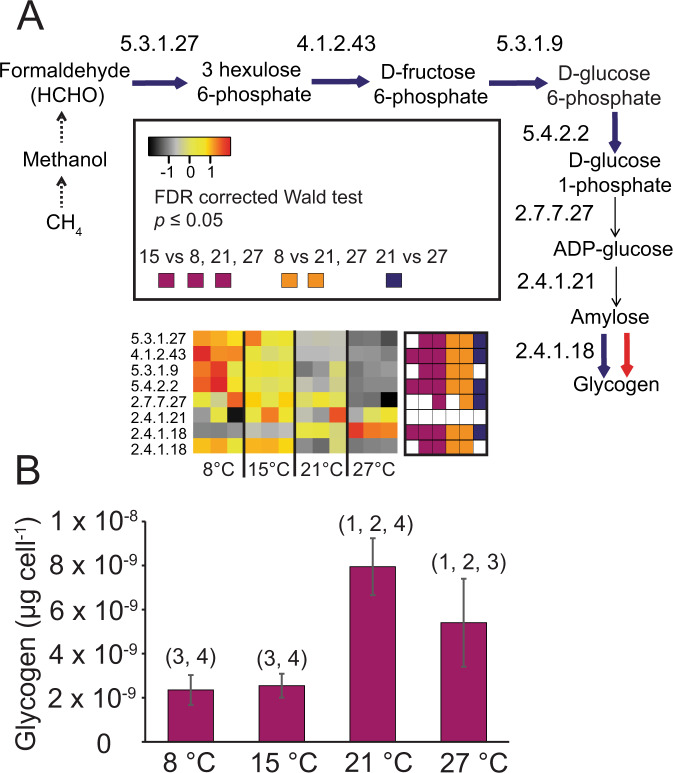
Glycogen synthesis and storage in *M. tundripaludum* SV96^T^. Gene expression for glycogen synthesis (**A**), and glycogen accumulation per cell (**B**) in *M. tundripaludum* SV96^T^. Heatmaps (**A**) indicate changes in the relative abundance of transcripts in the transcriptomes at 8, 15, 21, and 27 °C. The color scale reflects *z*-score normalized relative abundances, with black being lowest relative abundance, followed by gray, yellow, orange, and red, corresponding to increasing relative abundances. Significant differences (*p* < 0.05) in transcript relative abundances are displayed in square plots to the right of each heatmap, purple indicating significant difference between 15 °C and 8, 21 and 27 °C, respectively, when moving from left to right. Orange indicates significant differences between 8 °C and 21 and 27 °C. Dark blue indicates significant differences between 21 and 27 °C. All *p* values are estimated with a Wald test, implemented in DESeq2, and corrected for multiple testing using Benjamini Hochberg correction which adjusts for the false discovery rate. Blue arrows indicate that at least one of the genes encoding the enzyme responsible for catalyzing the reaction represented by this pathway step is significantly higher expressed at 8 °C than at two or three of the other temperatures. Red arrow indicates significantly higher expression at 27 °C. All adjusted *p* values, normalized counts, genome IDs (a unique identifier that allows tracking of each gene to the genome annotation available in https://mage.genoscope.cns.fr/microscope) and full protein names are given in SD C: Table C6. Bar graph (**B**) show the µg glycogen per cell in exponential state culture, estimated from a cell lysate with a known cell number. Considering the possibility of incomplete lysis, the numbers are likely conservative estimates. Error bars indicate standard deviation. Numbers above bars indicate to which of the other bars there are significant differences (*p* < 0.05).

Several bacteria are known to store superfluous carbon and energy as, for example, glycogen or PHA [64], and temperature effects on storage have been observed previously [37]. In searching the transcriptome of *M. tundripaludum* SV96^T^ for carbon storage pathway genes we identified all steps leading from CH_4_ oxidation, via the initial steps of RuMP-FBP (Fig. 3), to glycogen synthesis (Fig. 5A, SD C: Table C6). Starting from formaldehyde, the genes for six out of the eight steps needed to produce glycogen were upregulated at 8 °C and 15 °C (Fig. 5A). However, for the final step from amylose to glycogen, catalyzed by a 1,4-alpha-glucan branching enzyme (EC: 2.4.1.18), we counted two gene copies. These copies were expressed in opposite patterns in relation to temperature (Fig. 5A), suggesting this as an important regulatory step for glycogen synthesis. This also made us question whether the expression patterns really meant that glycogen synthesis is upregulated at low temperature. Thus, in a follow-up experiment, cells were harvested in exponential phase (the same physiological state of cells harvested for transcriptomics) for measurement of cellular glycogen. We could show that cells had accumulated highest concentrations of glycogen at 21 °C, followed by 27, 15, and 8 °C (Fig. 5B, SD B: Table B14). Thus, our measurements matched the transcriptional pattern of only one of the gene copies encoding a 1,4-alpha-glucan branching enzyme, not the overall expression pattern of the genes in the pathway (Fig. 5A).

### Regulatory adjustments of growth, membranes, cell walls, and exopolysaccharides

After having observed the adjustment of several central cellular mechanisms to different temperatures we surveyed those functions most directly related to growth. FtsZ is a key cytoskeleton protein involved in bacterial cell division, forming the Z-ring, a constricting structure at the division site [65, 66]. The relative gene expression pattern for FtsZ and related proteins reflected the growth rates of the cultures at different temperatures with similar expression levels at 15 and 21 °C, and lower expression at 8 and 27 °C (Fig. S11, SD C: Table C7). This further corresponded to the upregulation of genes for cell wall synthesis at both 15 and 21 °C (Fig. S12A, SD C: Table C8). At 27 °C we observed a strong upregulation of genes for exopolysaccharides (Fig. S12A, SD C: Table C8). As *M. tundripaludum* SV96^T^ does not grow above 30 °C [47], these responses might reflect adjustments needed for survival at high temperatures. Increased expression of exopolysaccharides is often associated with sub-optimal conditions, including temperatures outside the growth optimum range [67, 68] and can have a role as cryoprotectant in microorganisms [69]. Exopolysaccharides are also thought to protect cells from high temperatures by strengthening their structural integrity [69]. On the contrary, desaturation of fatty acids to increase membrane fluidity is a common response in bacteria exposed to low temperature, for example, [31, 70]. Correspondingly, we observed significant upregulation of fatty acid desaturase (*desA*) at 8 °C and 15 °C, relative to 21 and 27 °C (Fig. S12B, SD C: Table C8).

### Implications of thermal acclimation

In this study, we have demonstrated different thermal acclimation strategies in three members of the widespread methanotroph genus *Methylobacter*. We have also shown that the effect of temperature on growth and CH_4_ consumption depends on the CH_4_ concentration, and that CH_4_ oxidation rates do not correlate with temperature and growth. This means that higher temperatures can, in some instances, lead to more efficiently growing methanotrophs that oxidize less CH_4_ per cell over time. If the growth of such methanotrophs should be further inhibited by for example nutrient or oxygen limitations, increasing temperatures could lead to lower CH_4_ oxidation rates in some ecosystems, despite the microorganisms themselves being better adapted to the higher temperature. These counter-intuitive insights may explain why temperature effects on CH_4_ oxidation rates in nature vary so much. Furthermore, we have provided insights into the physiological adjustments that underlie thermal acclimation of CH_4_ oxidation and growth, focusing on *M. tundripaludum* SV96^T^. These physiological adjustments include changes in cell sizes, the protein biosynthesis machinery, glycogen storage, cell division, and exopolysaccharide expression.

Considering the scale of temperature change globally, including daily, seasonal, and long-term climate change, thermal acclimation of methanotrophs might exert considerable influence on global CH_4_ cycling. We show that the temperature effect on CH_4_ consumption is directly related to how different strains adjust their physiology during thermal acclimation. Furthermore, large differences in thermal acclimation strategies and temperature effects on CH_4_ consumption between strains suggest that also community shifts could have a substantial impact on the size of the biological CH_4_ sink.

## Materials and methods

### Cultures

During stock culture maintenance, pre-incubations, and experiments, *M. tundripaludum* SV96^T^ [47], *Methylobacter* sp. G7 (unpublished), and *Methylobacter luteus* ACM 3304 ^T^ [71] were cultivated in nitrate minimal salt (NMS) medium at pH 6.8 [72] (see [73] for trace element solution). See supplementary information (SI) methods section, subsection “Cultures” for more information.

### Acclimation

During acclimation, cells were incubated in NMS medium under ~80% air and ~20% CH_4_ (100% CH_4_ was injected into the air-containing bottle headspace) at 8, 15, 21, and 27 °C (*M. tundripaludum* SV96^T^ and *M. luteus* ACM 3304 ^T^), or 4, 8, 15 and 21 °C (*Methylobacter* sp. G7) (Fig. S2). The cultures were always allowed acclimation for the time needed to surpass 10 generations (7–12 days). During acclimation and experiments, cultures were shaken in a horizontal position, at 50 rpm. Some control experiments were carried out at 150 rpm to test the effect on mass transfer limitations. See SI methods, subsection “Acclimation” for more information.

### Shaking speed and mass transfer limitations

Strains that consume gases can experience mass transfer limitations due to low substrate solubility. See SI methods, subsection “Shaking speed and mass transfer limitations.” for more information on how we accounted for mass transfer limitation.

### Cell growth experiments

From samples of acclimated exponential phase *M. tundripaludum* SV96^T^ cultures (75 ml) with densities of ~1–3 × 10^7^ cells mL^−1^ 22 mL were transferred to three 125 mL glass bottles per temperature. Bottles were then prepared with headspaces of 80% air and 20% CH_4_ and a pressure of 1.3 atm. This resulted in 0.36–0.51 mM dissolved CH_4_, depending on the temperature, above the threshold of CH_4_ saturation for *M. tundripaludum* SV96^T^ at this density (0.1 mM CH_4_; Fig. S4B). The bottles were incubated at 8, 15, 21, and 27 °C and 50 rpm (Fig. S2A) or 150 rpm (Fig. S2E) for up to 36 h. See SD B: Table B11, B16 for incubation times and sampling time points for each experiment. At time zero and respective sampling intervals, 300 µL of cell suspension (in duplicate) was transferred to a Nunclon Delta Surface plate (Thermo Scientific, Waltham, MA, USA) and the optical density was measured (Spectra Max 250 microplate reader, Molecular Devices, San José, CA, USA) at 600 nm (diluted NMS medium as blank). For optical density measurements (OD_600_), blanks were subtracted from measurements.

### CH_4_ oxidation kinetics and growth kinetics

Subsamples of acclimated exponential-phase cultures with densities of 5 × 10^7^ cells mL^−1^ were aliquoted (21.6 mL) in 125 mL glass bottles for measurement of CH_4_ oxidation and growth rates at seven different CH_4_ concentrations and four different temperatures (8, 15, 21, and 27 °C for *M. luteus* ACM 3304 ^T^ and *M. tundripaludum* SV96^T^, and 4, 8, 15, and 21 °C for *Methylobacter* sp. G7). We used three aliquots for each of the seven CH_4_ concentrations, for a total of 21 bottles per temperature. With four temperatures, that amounted to a total of 84 bottles per strain (Fig. S2A, B, C, F, and G). For each temperature, two negative controls (medium without cells) in 125 mL glass bottles were prepared per CH_4_ concentration (56 negative controls in total per strain). None of the negative controls indicated non-biological CH_4_ consumption or leakage. To create the seven different CH_4_ concentrations, seven volumes (200 µL; 600 µL; 1,5 mL; 3 mL; 6 mL; 12 mL; 15 mL) of 100% CH_4_ were injected with a plastic syringe (BD Plastipak, Franklin Lakes, NJ, USA) with a sterile 0.5 × 16 mm needle (BD Microlance, Franklin Lakes, NJ, USA) from a multi-layer polypropylene gas bag (RESTEK, Bad Homburd vor der Höhe, Germany). Final headspace concentrations of CH_4_ ranged between 1000 and 130,000 p.p.m.v. while dissolved concentrations of CH_4_ ranged between 1.5 and 260 µM depending also on temperature. The gas pressures in the bottles were then adjusted to a total headspace pressure of ~1.3 atm at 20 °C by injecting additional volumes of air. See SI methods, subsection “CH_4_ oxidation kinetics and growth kinetics” for more information.

### Glycogen quantification

The cells were acclimated and cultivated under 20% CH_4_ in air as described above. Five replicate bottles with 20 mL culture and 20% CH_4_ in air were incubated per temperature in darkness with 50 rpm shaking, five bottles with a starting cell concentration of ~1 × 10^7^ cells mL^−1^ and five with a density of ~1 × 10^8^ cells mL^-1^. An incubation length of 48 h at 15 and 21 °C was sufficient to reach high-density exponential growth phase at 27 °C and 8 °C it took 60 and 90 h, respectively, to reach those same cell densities. During harvest, 2 mL of culture was sampled for glycogen measurements from the dense cultures and 10 mL from the dilute cultures. The samples were processed according to recommendations from the manufacturer (Glycogen assay kit, ab65620, Abcam, Cambridge, UK). Glycogen was quantified using a plate reader (GloMax Explorer, Promega, Madison, WI, USA). See SI methods, subsection “Glycogen quantification” for more information.

### OD600 to cell number conversion and cell sizes

To normalize rates to cell numbers, standard curves correlating optical density (OD_600_) to cell numbers were created for the three strains: *M. tundripaludum* SV96^T^, *M. luteus* ACM 3304 ^T^ and *Methylobacter* sp. G7 For size estimation, cells were visualized using a Carl Zeiss AxioObserver Z1 with a 100x objective and Bright-Field and measured with the size estimation tool available in the AxioVision SE64 Rel 4.9.1 software. Cultures for size estimation were prepared by acclimation of cultures to 8 and 21 °C, as described above, followed by cultivation to reach exponential phase and harvest for size estimation. See SI methods, subsection “OD600 to cell number conversion and cell sizes” for more information.

### Calculations

Specific growth rates were calculated as the slope of the natural logarithm of optical densities against time, during exponential growth. Mixing ratios of CH_4_ and CO_2_ were calculated by comparison to certified standards. Masses of headspace and dissolved CH_4_ and total CO_2_ at different temperatures were calculated from the mixing ratios of CH_4_ and CO_2_ using Henry’s Law, assuming an ideal state, knowing the ambient pressure, temperature, headspace volume of the bottle, headspace pressure, liquid volume, and respective temperature-dependent solubility constants of the gases. All calculations accounted for removal of gas and liquid for measurements. We calculated the CH_4_ oxidation and CO_2_ production rates from slopes of linear models fitted to the concentrations measured during experiments and adjusted to the number of cells in the culture. Growth efficiencies were calculated by multiplying the specific growth (cell divisions cell^−1^ h^−1^) rate by 10^8^ and then dividing by CH_4_ the oxidation rates (µmol CH_4_ oxidized 10^8^ cells^−1^ h^−1^) to give cell divisions per µmol CH_4_ oxidized. The data used to estimate growth efficiency were the values predicted for each measurement time-point from the non-linear Michaelis-Menten regression models. For estimates of specific growth rates, CH_4_ oxidation, and growth efficiency at CH_4_ saturation, V_max(app)_ values predicted from the Michaelis-Menten kinetics models were used (see below for V_max(app)_ estimation). See SI methods, subsection “Calculations” for more information.

### Statistics for physiological data

See SI methods, subsection “Statistics for physiological data” for a complete description of the methods used.

### RNA and DNA extraction, and sequencing

Six cultures of *M. tundripaludum* SV96^T^ were acclimated at each of the temperatures, 8, 15, 21, or 27 °C, as described above. After acclimation, the cultures were cultivated to a sufficient cell density for extraction in exponential phase growth. Cultures at 8 and 27 °C were incubated for 36 h, and cultures at 15 and 21 °C for 16 h, prior to harvest for RNA and DNA extractions. See SI methods, subsection “RNA and DNA extraction, and sequencing” for more information.

### Computational analyses

See SI methods, subsection “Computational analyses” for a complete description of the methods used to analyze the transcriptomes.

### Maps

See SI methods, subsection “Maps” for a complete description of the methods used.

## Supplementary information

Supplementary Information

Supplementary dataset A

Supplementary dataset B

Supplementary dataset C

## Data Availability

The RNA-seq data from this study have been deposited under the accession project number PRJNA390985 in the NCBI short read archive (SRA). The individual experiment identification numbers for each of the 12 datasets are found in SD B: Table B13. Scripts for RNA-seq pre-processing, gene expression analysis, statistical tests, modelling, and visualization in R, are provided online (“Thermal acclimation of methanotrophs from the genus Methylobacter”, 10.18710/Z1QF8W, DataverseNO). Data for maps are provided in SD A. Physiological data are provided in SD B. The processed and normalized transcript counts and statistics for the differential gene expression analyses are provided in SD C.
