# Supplementary material for: Thermal acclimation of methanotrophs from the genus Methylobacter
Source: ISME J. 2023 Jan 18;17(4):502–13. doi: 10.1038/s41396-023-01363-7 (PMC10030640; doi:10.1038/s41396-023-01363-7)
Supplement: Supplementary file 1 — Supplementary Information [file 41396_2023_1363_MOESM1_ESM.pdf]

Supplementary information (SI) for:

Thermal acclimation of methanotrophs from the genus *Methylobacter*

Alexander T. Tveit[a*], Andrea Söllinger[a], Edda Marie Rainer[a], Alena Didriksen[a], Anne Grethe Hestnes[a], Liabo Motleleng[a], Hans-Jörg Hellinger[bc], Thomas Rattei[b], Mette M. Svenning[a]

[a]*Department of Arctic and Marine Biology, UiT The Arctic University of Norway, Tromsø, Norway.*

[b]*University of Vienna, Centre for Microbiology and Environmental Systems Science, Vienna, Austria.*

[c]*University of Vienna, Doctoral School in Microbiology and Environmental Science, Vienna, Austria.*

*Corresponding author: Alexander Tøsdal Tveit

alexander.t.tveit@uit.no

Content:

Supplementary Figures: pages 2 – 19

Supplementary materials and methods: pages 19 – 31

Supplementary figures

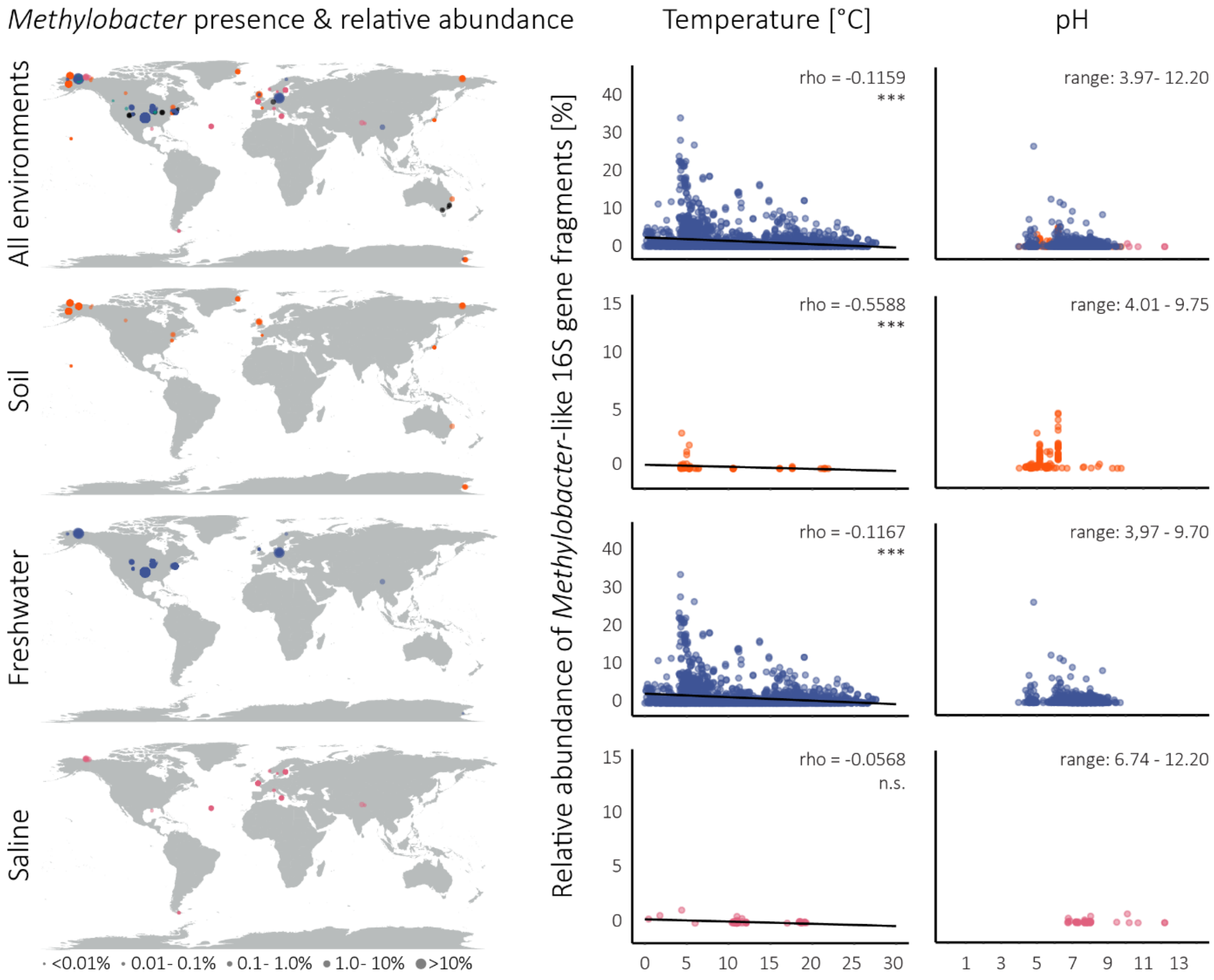

Figure S1. Global distribution of *Methylobacter*. Presence and relative abundances of *Methylobacter* 16S rRNA gene fragments detected in Earth Microbiome Project (EMP) samples derived from various environments (SD A: Table A2). Relative abundances are represented by five categories (<0.01%, 0.01 – 0.1%, 0.1 – 1.0%, 1.0 – 10%, and >10%). Scatter plots show relative abundances of *Methylobacter* 16S rRNA gene fragments depending on temperature and pH (see SD A: Table A5 for details on the correlations between relative abundances and temperature).

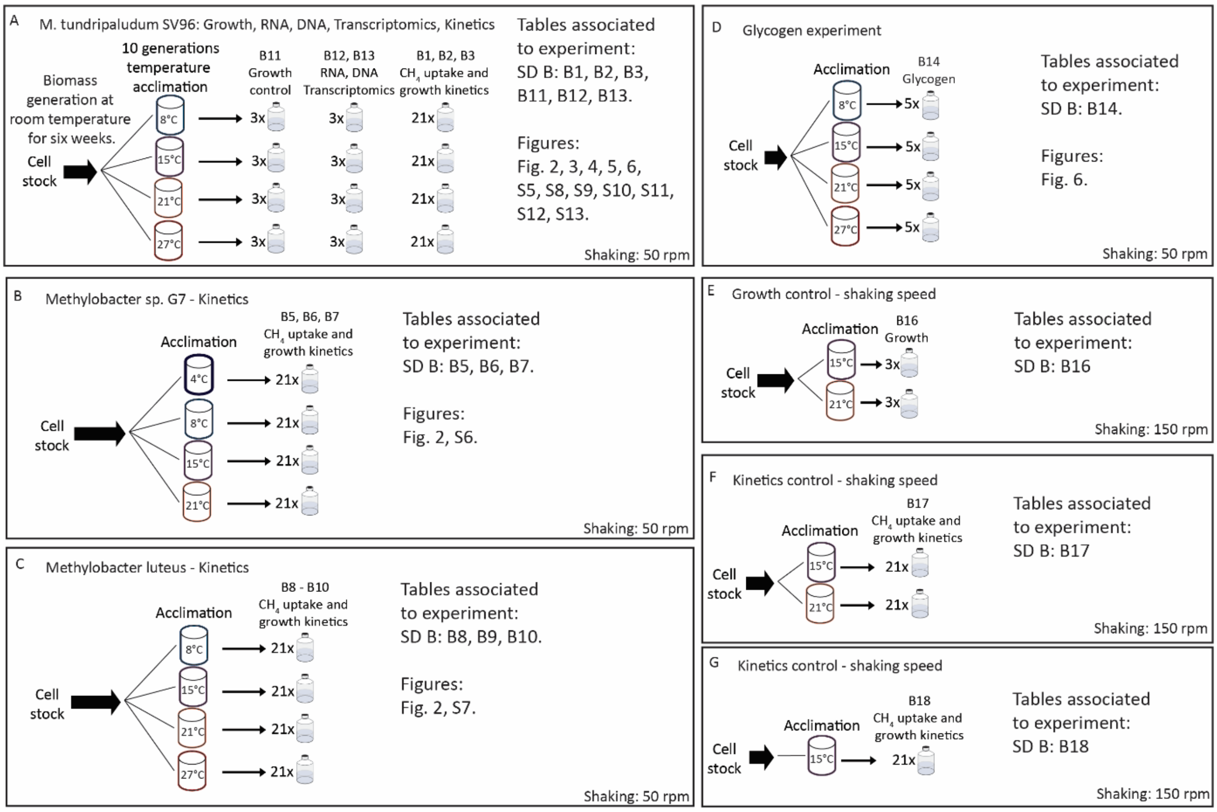

Figure S2. Experimental setup. Each box indicates an independent experiment. “Cell stock” represent a frozen cell culture as starting point for cultivation. Thermal acclimation times varied due to the different growth rates at different temperatures. We standardized it to approximately 10 generations of acclimation. Numbers in front of bottles indicate the number of replicates. In the case of kinetics, it indicates the number of replicates multiplied by the number of CH_4 concentrations (3 × 7). The corresponding dataset(s) and figures to each experiment are indicated in each box, the numbers B1 – B18 referring to the individual tables in Supplementary dataset (SD) B.

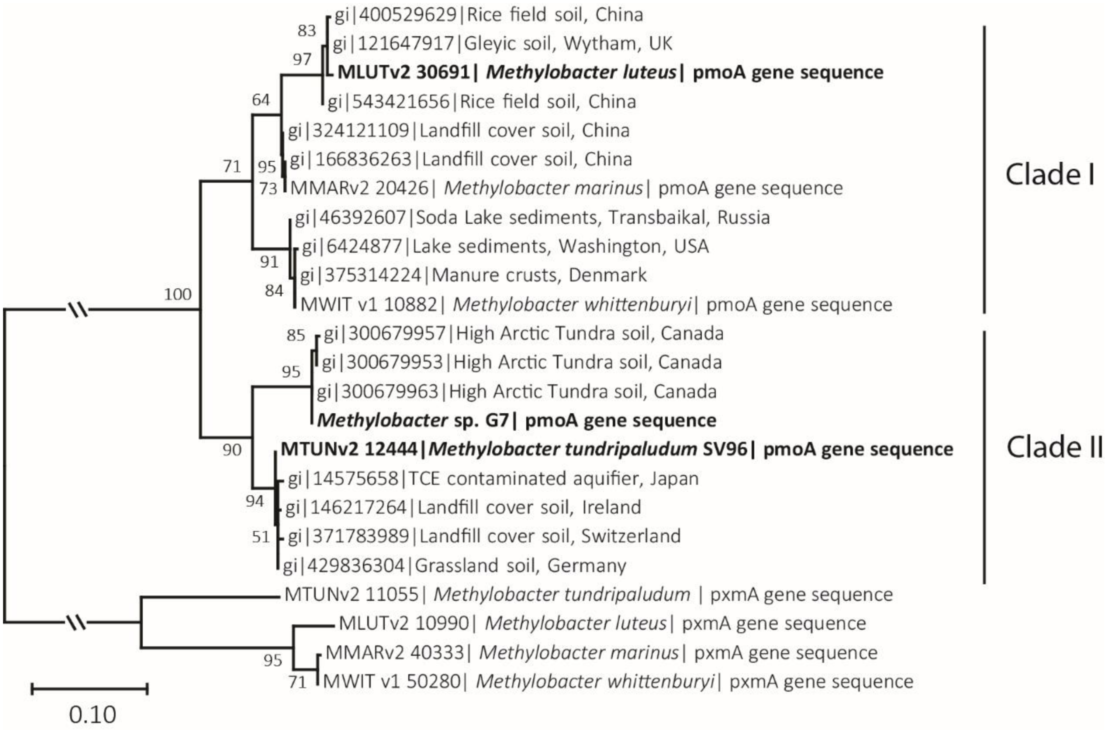

Figure S3. Phylogenetic tree of *pmoA* sequences belonging within the genus *Methylobacter*. Sequence names include gene identifier, ecosystem type of origin, and country of origin. The evolutionary history was inferred by using the Maximum Likelihood method and Kimura 2-parameter model (24). The tree with the highest log likelihood (-2368.76) is shown. The percentage of trees in which the associated taxa clustered together is shown next to the branches. Initial tree(s) for the heuristic search were obtained automatically by applying Neighbor-Join and BioNJ algorithms to a matrix of pairwise distances estimated using the Maximum Composite Likelihood (MCL) approach, and then selecting the topology with superior log likelihood value. The tree is drawn to scale, with branch lengths measured in the number of substitutions per site. This analysis involved 24 nucleotide sequences. Codon positions included were 1st+2nd+3rd+Noncoding. There was a total of 471 positions in the final dataset. Evolutionary analyses were conducted in MEGA X (25). See (22) for more detailed information about the recent clade organization of *Methylobacter*. As an outgroup we have used *pxmA* genes encoding close relatives of the *pmoA* gene, but with an unknown function.

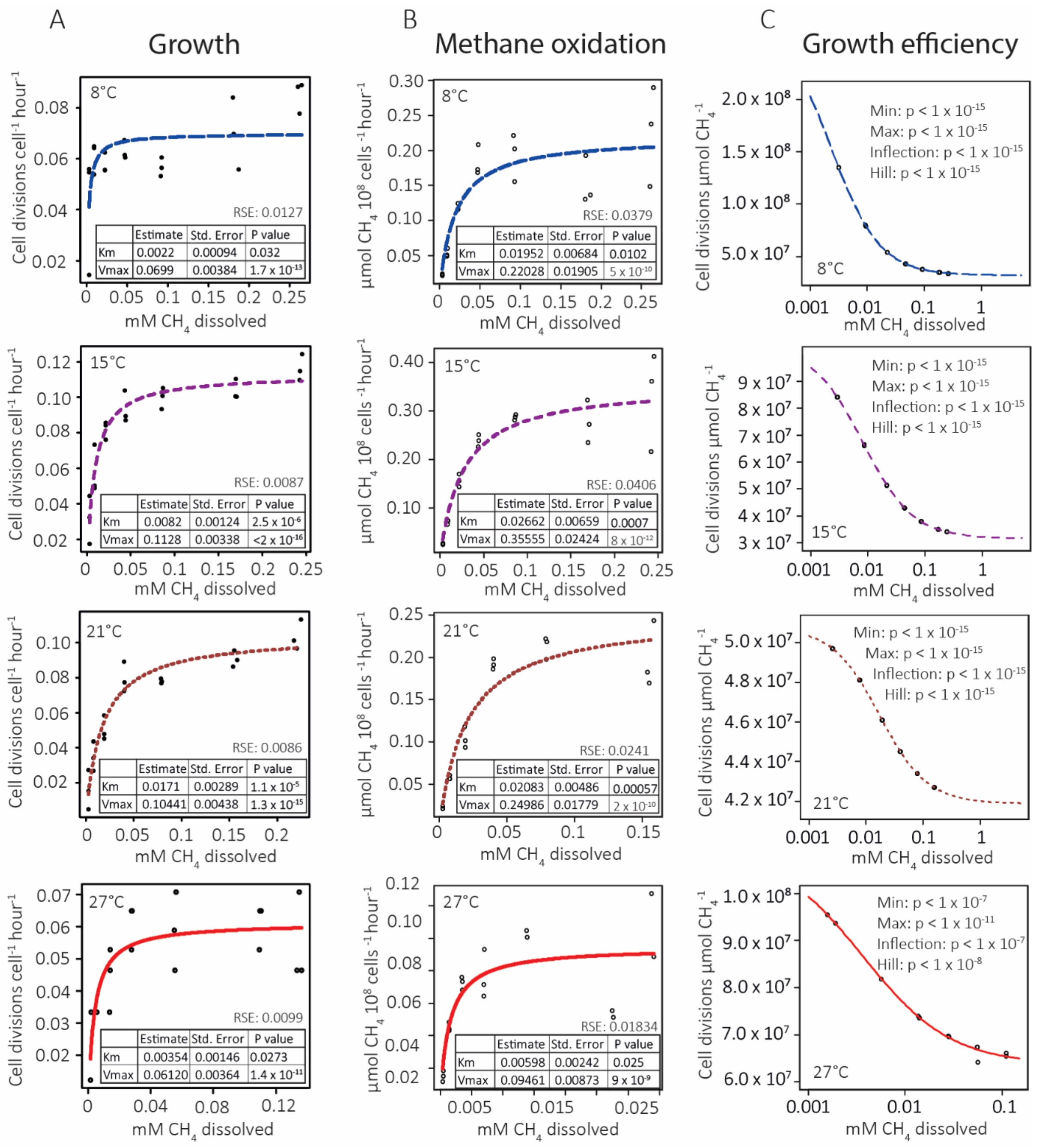

Figure S4. Growth kinetics, CH_4 oxidation kinetics and growth efficiency at different temperatures for *M. tundripaludum* SV96^T. Specific growth rates (cell divisions per cell per hour) are shown for different dissolved CH_4 concentrations at different temperatures in **(A)**. The x-axis is on a log10 scale to better resolve the differences between temperatures in the low and high end of concentrations. CH_4 oxidation rates per cell for different dissolved CH_4 concentrations at different temperatures are shown in **(B)**. $K_{m(app)}$, $V_{max(app)}$, standard errors and p-values for the coefficient estimates, and residual standard errors of the models were estimated using non-linear regression, applying a Michaelis-Menten function (see the Materials and Methods section "Statistics for

physiological measurements”). Growth efficiency (cell divisions per µmol CH_4 oxidized) at different dissolved CH_4 concentrations and temperatures are shown in **(C)**. These growth efficiency estimates were calculated from the predicted rates in **(A)** and **(B)** by dividing specific growth rate predictions by CH_4 oxidation rate predictions to obtain predictions of cell divisions per µmol CH_4 oxidized. Four-parameter logistics curves (dose-response model) were fitted to the resulting quotients. The p-values for the four parameters (min, max, inflection and hill) of the dose-response models for different temperatures are shown inside panels in **(C)**. In all panels, the x-axis is displayed on a log10 scale. The corresponding data can be found in SD B: Tables B1, B2 and B3.

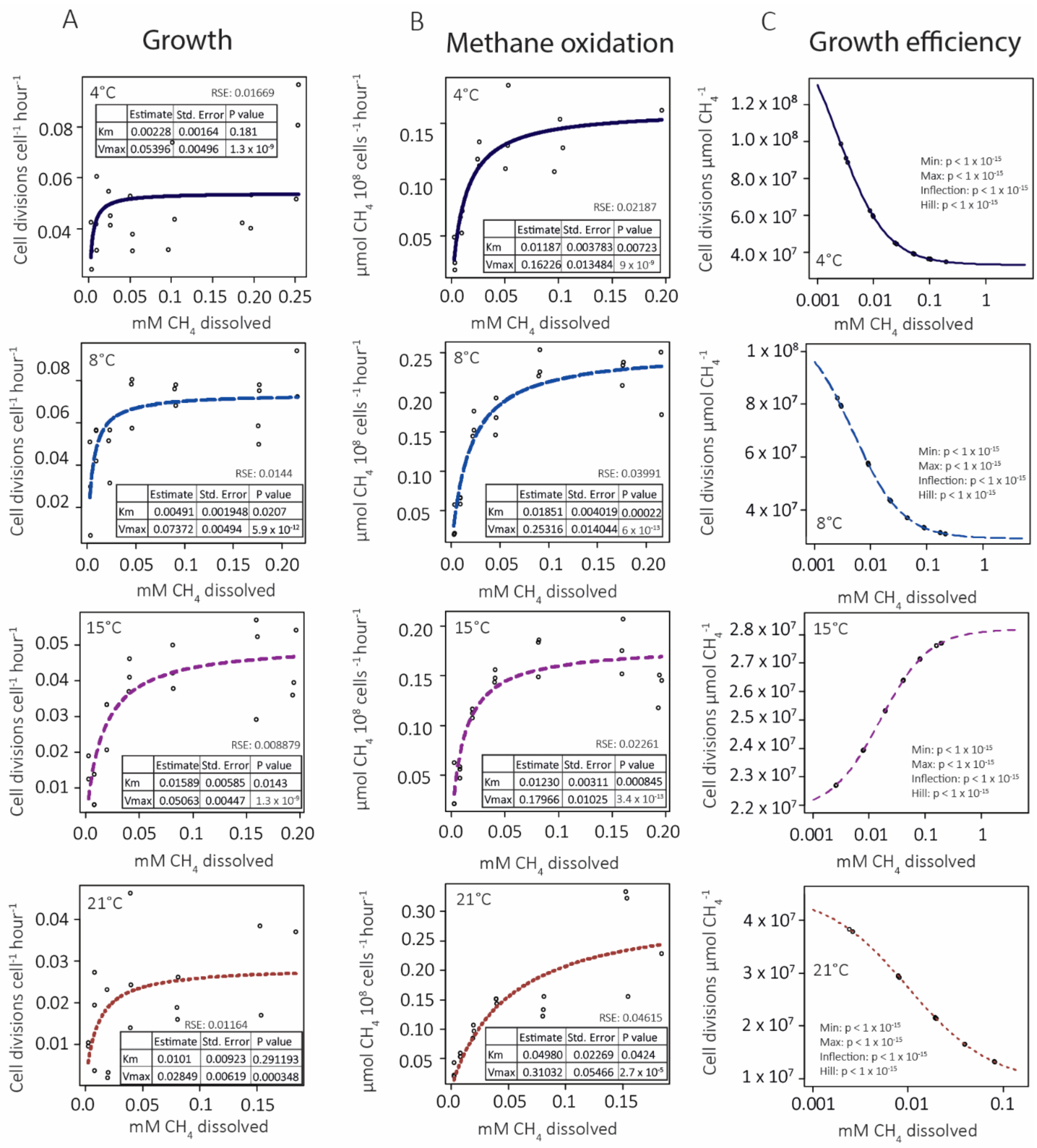

Figure S5. Growth kinetics, CH_4 oxidation kinetics and growth efficiency at different temperatures for *Methylobacter* sp. G7. Specific growth rates (cell divisions per cell per hour) are shown for different dissolved CH_4 concentrations at different temperatures in **(A)**. The x-axis is on a log10 scale to better resolve the differences between temperatures in the low and high end of concentrations. CH_4 oxidation rates per cell for different dissolved CH_4 concentrations at different temperatures are shown in **(B)**. $K_{m(app)}$, $V_{max(app)}$, standard errors and p-values for the coefficient estimates, and residual standard errors of the models were estimated using non-linear regression, applying a Michaelis-Menten function (see the Materials and Methods section "Statistics for

physiological measurements”). Growth efficiency (cell divisions per µmol CH_4 oxidized) at different dissolved CH_4 concentrations and temperatures are shown in **(C)**. These growth efficiency estimates were calculated from the predicted rates in **(A)** and **(B)** by dividing specific growth rate predictions by CH_4 oxidation rate predictions to obtain predictions of cell divisions per µmol CH_4 oxidized. Four-parameter logistics curves (dose-response model) were fitted to the resulting quotients. The p-values for the four parameters (min, max, inflection and hill) of the dose-response models for different temperatures are shown inside panels in **(C)**. In all panels, the x-axis is displayed on a log10 scale. The corresponding data can be found in SD B: Tables B5, B6 and B7.

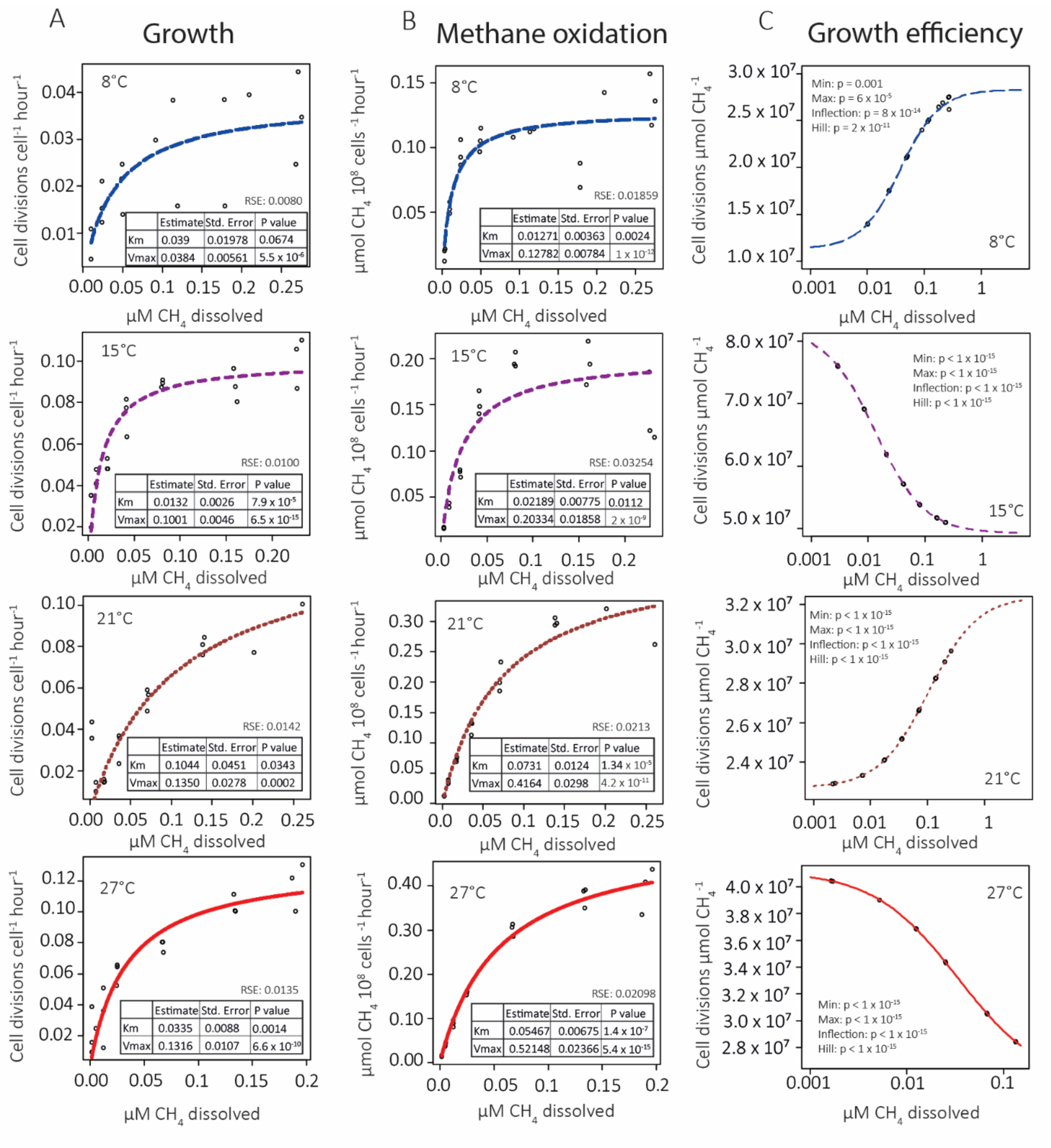

Figure S6. Growth kinetics, CH_4 oxidation kinetics and growth efficiency at different temperatures for *Methylobacter luteus* ACM 3304^T. Specific growth rates (cell divisions per cell per hour) are shown for different dissolved CH_4 concentrations at different temperatures in **(A)**. The x-axis is on a log10 scale to better resolve the differences between temperatures in the low and high end of concentrations. CH_4 oxidation rates per cell for different dissolved CH_4 concentrations at different temperatures are shown in **(B)**. $K_{m(app)}$, $V_{max(app)}$, standard errors and p-values for the coefficient estimates, and residual standard errors of the models were estimated using non-linear regression, applying a Michaelis-Menten function (see the Materials and Methods section “Statistics for physiological measurements”). Growth efficiency (cell divisions per µmol CH_4 oxidized) at different

dissolved CH_4 concentrations and temperatures are shown in **(C)**. These growth efficiency estimates were calculated from the predicted rates in **(A)** and **(B)** by dividing specific growth rate predictions by CH_4 oxidation rate predictions to obtain predictions of cell divisions per µmol CH_4 oxidized. Four-parameter logistics curves (dose-response model) were fitted to the resulting quotients. The p-values for the four parameters (min, max, inflection and hill) of the dose-response models for different temperatures are shown inside panels in **(C)**. In all panels, the x-axis is displayed on a log10 scale. The corresponding data can be found in SD B: Tables B8, B9 and B10.

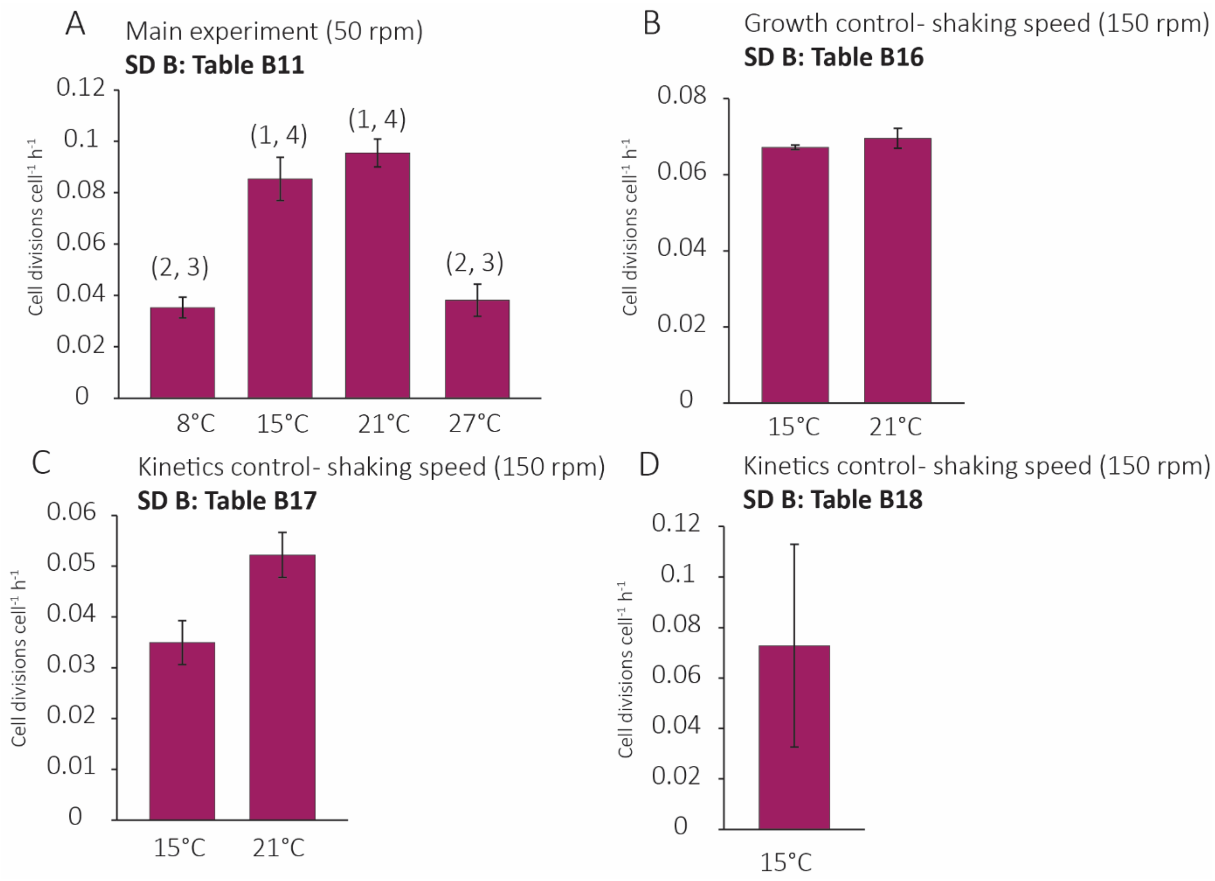

Figure S7. Specific growth rate experiments at 50 and 150 rpm shaking. Bar graphs show the specific growth rates of *M. tundripaludum* SV96^T at different temperatures, under a headspace of 20 % CH_4 (mix of CH_4 and air). The corresponding dataset to each experiment is indicated next to the title of each bar graph, referring to the individual tables in supplementary dataset B. For A and B: Significant differences (p.adjust < 0.05, multiple testing correction using the approach of Benjamini-Hochberg) to 8 °C (1), 15 °C (2), 21 °C (3) and 27 °C (4) are indicated with numbers above the individual bars. Error bars represent standard deviation. For C and D: Rates indicate $V_{max(app)}$ predicted from non-linear regression, applying a Michaelis-Menten function (see the Materials and Methods section "Statistics for physiological measurements"). Error bars indicate standard errors of the model predictions.

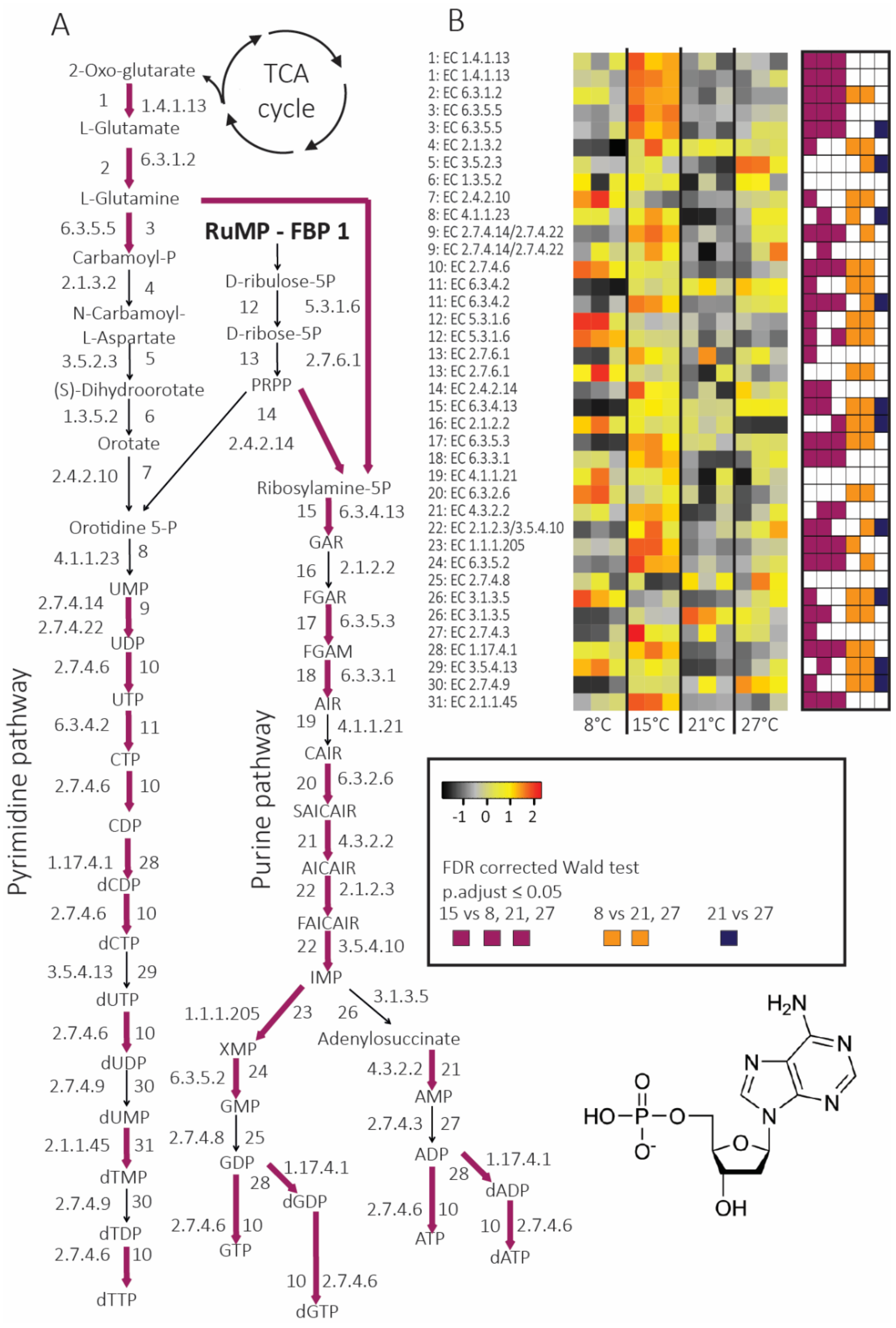

Figure S8. Gene transcription for nucleotide metabolism in *M. tundripaludum* SV96^{T}. Heatmaps indicate changes in the relative abundance of transcripts in the transcriptomes at 8, 15, 21 and 27 °C. The colour scale reflects z-score normalized relative abundances, with black being lowest relative abundance, followed by grey, yellow, orange and red, corresponding to increasing relative abundances. Significant differences (p.adjust < 0.05)

in relative transcript abundances are displayed in square plots to the right of each heatmap, purple indicating significant difference between 15 °C and 8, 21 and 27 °C, respectively, when moving from left to right. Orange indicates significant differences between 8 °C and 21 and 27 °C. Dark blue indicates significant differences between 21 and 27 °C. The p-values are estimated with a Wald test, implemented in DESeq2, and corrected for multiple testing using Benjamini-Hochberg correction which adjusts for the false discovery rate. Purple arrow indicates that at least one of the genes encoding the enzyme responsible for catalysing the reaction represented by this pathway step is significantly higher expressed at 15 °C than at two or three of the other temperatures. All adjusted p-values, normalized counts, genome IDs, full protein names and gene names are given in SD C: Table C2. Numbers next to heatmaps refer to corresponding genes in SD C: Table C2 and corresponding metabolic step illustrated in the figure.

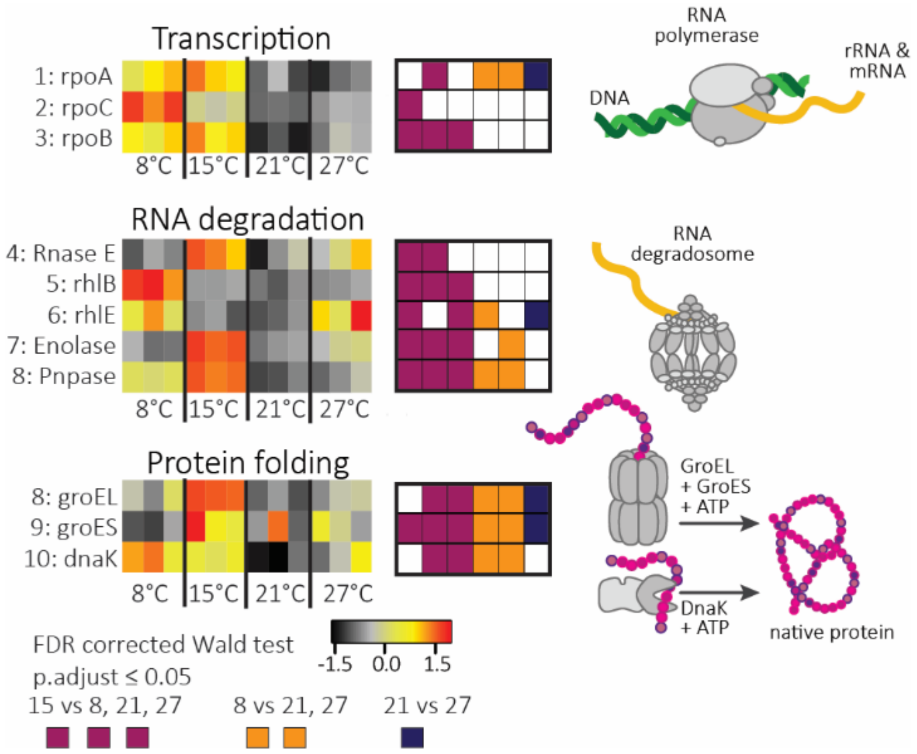

Figure S9. Gene transcription for RNA polymerase, RNA degradation and protein folding. Heatmaps indicate changes in the relative abundance of transcripts in the transcriptomes at 8, 15, 21 and 27 °C. The colour scale reflects z-score normalized relative abundances, with black being lowest relative abundance, followed by grey, yellow, orange, and red, corresponding to increasing relative abundances. Significant differences (p.adjust < 0.05) in relative transcript abundances are displayed in square plots to the right of each heatmap, purple indicating significant difference between 15 °C and 8, 21 and 27 °C, respectively, when moving from left to right. Orange indicates significant differences between 8 °C and 21 and 27 °C. Dark blue indicates significant differences between 21 and 27 °C. The p-values were estimated with a Wald test, implemented in DESeq2, and corrected for multiple testing using the approach of Benjamini-Hochberg, which adjusts for the false discovery rate. All adjusted p-values, normalized counts, genome IDs and full protein names are given in SD C: Table C3. The polymerase, degradosome and protein folding system cartoons are from a recent illustration (26). Numbers next to heatmaps refer to corresponding genes in SD C: Table C3 and corresponding metabolic step illustrated in the figure.

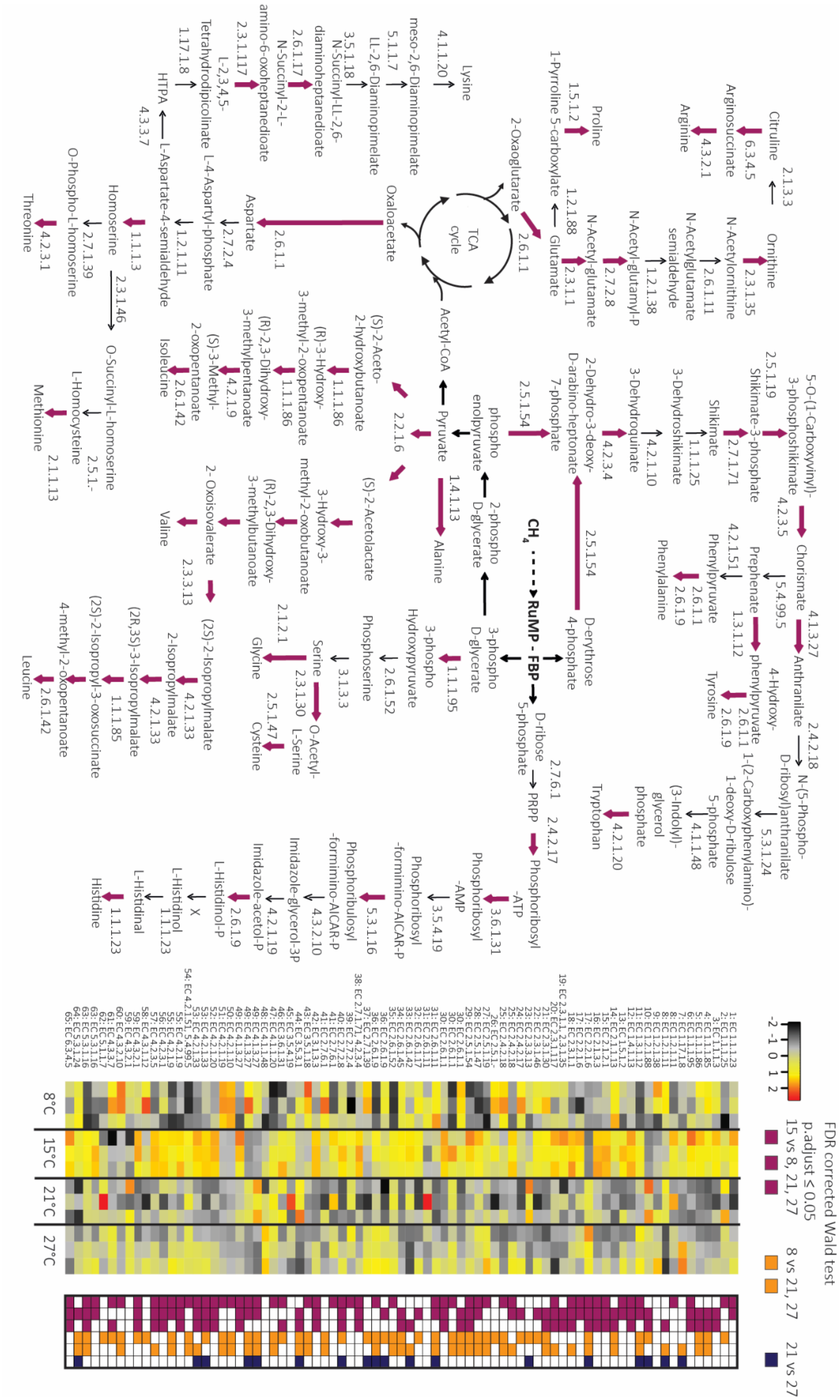
FDR corrected Wald test
p.adjust ≤ 0.05
15 vs 8, 21, 27
8 vs 21, 27
21 vs 27
8°C
15°C
21°C
27°C
TCA cycle
CH4 - - - ▶RuMP - FBP
Citruline
Ornithine
Arginosuccinate
Arginine
N-Acetylornithine
N-Acetylglutamate semialdehyde
N-Acetyl-glutamyl-P
N-Acetyl-glutamate
Proline
1-Pyrroline 5-carboxylate
Glutamate
2-Oxoglutarate
Lysine
meso-2,6-Diaminopimelate
LL-2,6-Diaminopimelate
N-Succinyl-LL-2,6-diaminoheptanedioate
N-Succinyl-2-L-amino-6-oxoheptanedioate
L-2,3,4,5-Tetrahydrodipicolinate
HTPA
L-4-Aspartyl-phosphate
L-Aspartate-4-semialdehyde
Aspartate
Oxaloacetate
Homoserine
O-Phospho-L-homoserine
Threonine
O-Succinyl-L-homoserine
L-Homocysteine
Methionine
Acetyl-CoA
Pyruvate
Alanine
phospho enolpyruvate
2-phospho D-glycerate
3-phospho D-glycerate
3-phospho Hydroxypyruvate
Phosphoserine
Serine
O-Acetyl-L-Serine
Cysteine
Glycine
(S)-2-Aceto-2-hydroxybutanoate
(R)-3-Hydroxy-3-methyl-2-oxopentanoate
(R)-2,3-Dihydroxy-3-methylpentanoate
(S)-3-Methyl-2-oxopentanoate
Isoleucine
(S)-2-Acetolactate
3-Hydroxy-3-methyl-2-oxobutanoate
(R)-2,3-Dihydroxy-3-methylbutanoate
2-Oxoisovalerate
Valine
(2S)-2-Isopropylmalate
2-Isopropylmalate
(2R,3S)-3-Isopropylmalate
(2S)-2-Isopropyl-3-oxosuccinate
4-methyl-2-oxopentanoate
Leucine
D-arabino-heptonate 7-phosphate
2-Dehydro-3-deoxy-
3-Dehydroquinate
3-Dehydroshikimate
Shikimate
Shikimate-3-phosphate
5-O-(1-Carboxyvinyl)-3-phosphoshikimate
Chorismate
Prephenate
Phenylpyruvate
Phenylalanine
4-Hydroxy-phenylpyruvate
Tyrosine
Anthranilate
N-(5-Phospho-D-ribosyl)anthranilate
1-(2-Carboxyphenylamino)-1-deoxy-D-ribulose 5-phosphate
(3-Indolyl)-glycerol phosphate
Tryptophan
D-erythrose 4-phosphate
D-ribose 5-phosphate
PRPP
Phosphoribosyl -ATP
Phosphoribosyl -AMP
Phosphoribosyl -formimino-AICAR-P
Phosphoribulosyl -formimino-AICAR-P
Imidazole-glycerol-3P
Imidazole-acetol-P
L-Histidinol-P
L-Histidinol
L-Histidinal
Histidine

Figure S10. Gene transcription for amino acid metabolism in *M. tundripaludum* SV96^T. Heatmaps indicate changes in the relative abundance of transcripts in the transcriptomes at 8, 15, 21 and 27 °C. The colour scale reflects z-score normalized relative abundances, with black being lowest relative abundance, followed by grey, yellow, orange, and red, corresponding to increasing relative abundances. Significant differences (p.adjust < 0.05) in relative transcript abundances are displayed in square plots to the right of each heatmap, purple indicating significant difference between 15 °C and 8, 21 and 27 °C, respectively, when moving from left to right. Orange indicates significant differences between 8 °C and 21 and 27 °C. Dark blue indicates significant differences between 21 and 27 °C. The p-values are estimated with a Wald test, implemented in DESeq2, and corrected for multiple testing using the approach of Benjamini-Hochberg which adjusts for the false discovery rate. Purple arrow indicates that at least one of the genes encoding the enzyme responsible for catalysing the reaction represented by this pathway step is significantly higher expressed at 15 °C than at two or three of the other temperatures. All adjusted p-values, normalized counts, genome IDs, gene names and full protein names are given in SD C: Table C5. Numbers next to heatmaps refer to corresponding genes in SD C: Table C5 and corresponding metabolic step illustrated in the figure.

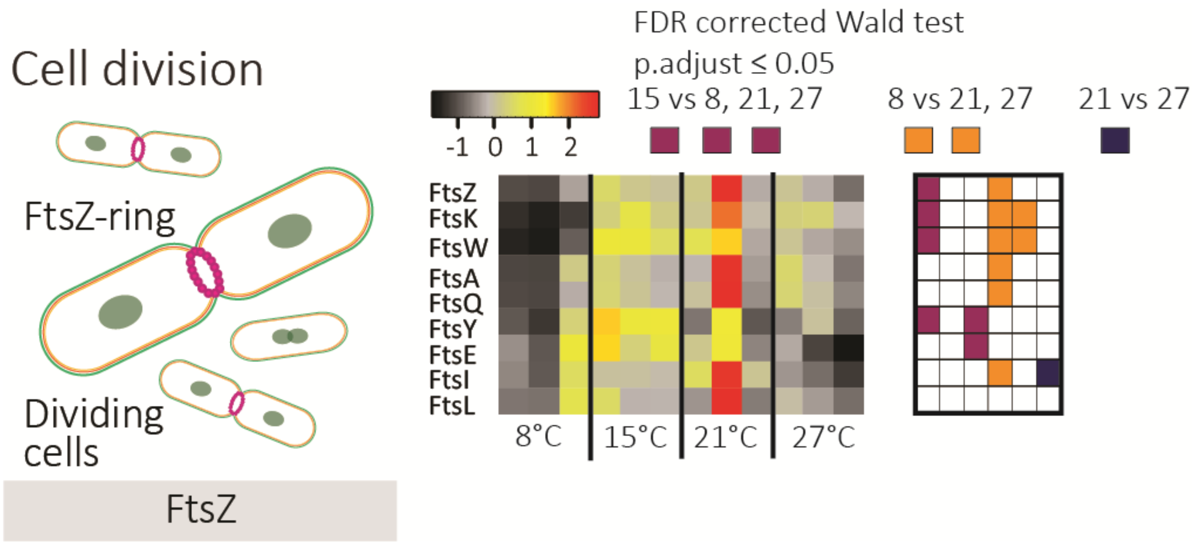

Figure S11. Gene transcription for cell division in *M. tundripaludum* SV96ᵀ. Heatmaps indicate changes in the relative abundance of transcripts in the transcriptomes at 8, 15, 21 and 27 °C. The colour scale reflects z-score normalized relative abundances, with black being lowest relative abundance, followed by grey, yellow, orange, and red, corresponding to increasing relative abundances. Significant differences (p.adjust < 0.05) in relative transcript abundances are displayed in square plots to the right of each heatmap, purple indicating significant difference between 15 °C and 8, 21 and 27 °C, respectively, when moving from left to right. Orange indicates significant differences between 8 °C and 21 and 27 °C. Dark blue indicates significant differences between 21 and 27 °C. The p-values are estimated with a Wald test, implemented in DESeq2, and corrected for multiple testing using the approach of Benjamini-Hochberg which adjusts for the false discovery rate. All adjusted p-values, normalized counts, genome IDs, gene names and full protein names are given in SD C: Table C7. The cell division cartoon is from a recent illustration (26).

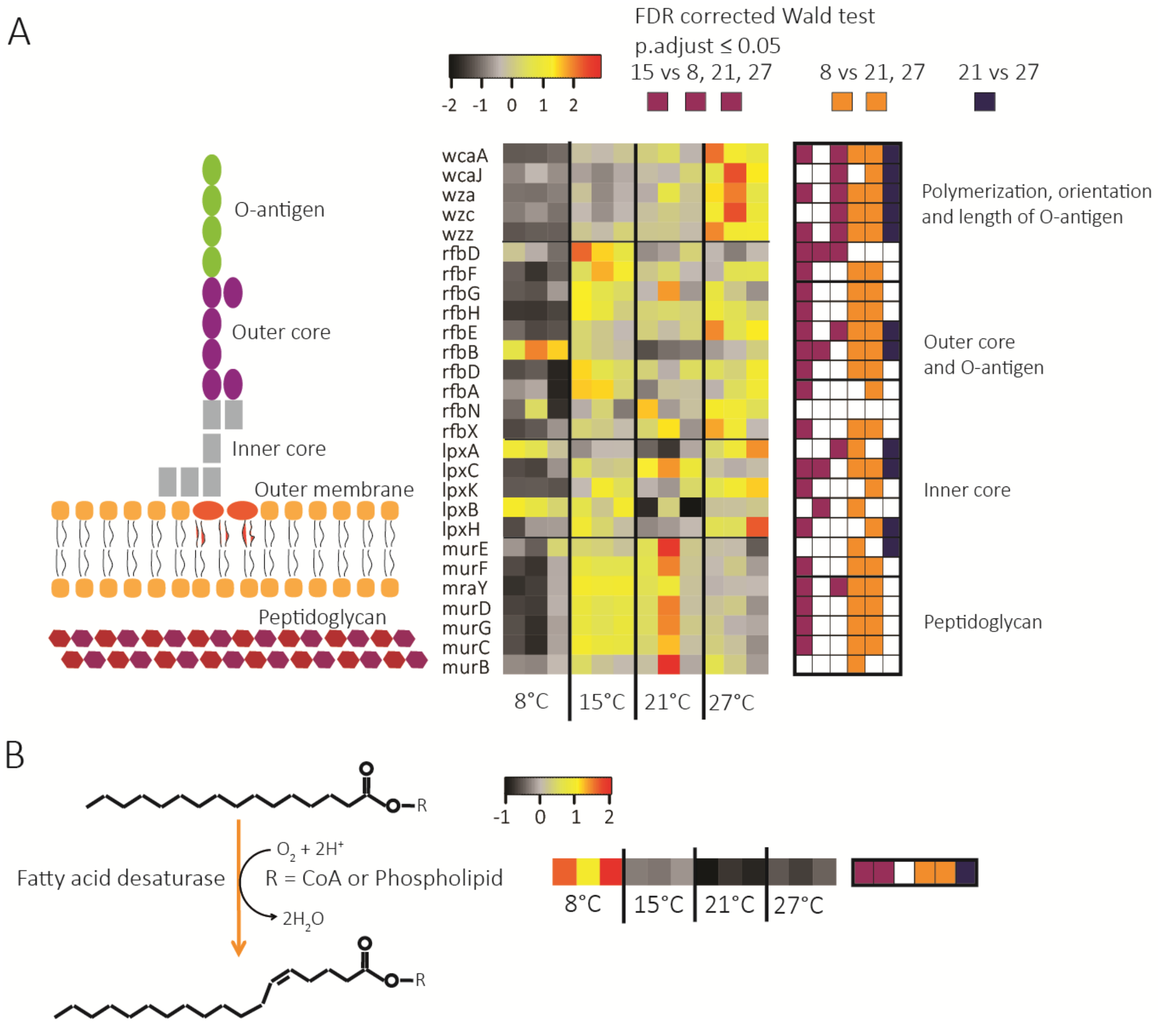

Figure S12. Gene transcription for cell wall synthesis, exopolysaccharides, and fatty acid desaturation in *M. tundripaludum* SV96^T. (A) shows transcriptional patterns and significance tests for cell wall and exopolysaccharide synthesis. (B) shows fatty acid desaturase transcription and corresponding significance tests. Heatmaps indicate changes in the relative abundance of transcripts in the transcriptomes at 8, 15, 21 and 27 °C. The colour scale reflects z-score normalized relative abundances, with black being lowest relative abundance, followed by grey, yellow, orange, and red, corresponding to increasing relative abundances. Significant differences (p.adjust < 0.05) in relative transcript abundances are displayed in square plots to the right of each heatmap, purple indicating significant difference between 15 °C and 8, 21 and 27 °C, respectively, when moving from left to right. Orange indicates significant differences between 8 °C and 21 and 27 °C. Dark blue indicates significant differences between 21 and 27 °C. The p-values are estimated with a Wald test, implemented in DESeq2, and corrected for multiple testing using the approach of Benjamini-Hochberg which adjusts for the false discovery rate. Orange arrow in (B) indicates significantly higher relative abundance of transcripts at 8 °C than at

two or three of the other temperatures. All adjusted p-values, normalized counts, genome IDs, E.C. numbers, gene names and full protein names are given in SD C: Table C8.

Supplementary materials and methods

Cultures. During stock culture maintenance, pre-incubations, and experiments, *M. tundripaludum* SV96^T (1), *Methylobacter* sp. G7 (unpublished), and *Methylobacter luteus* ACM 3304^T (2) were cultivated in nitrate minimal salt (NMS) medium at pH 6.8 (3) (see (4) for trace element solution). We used 125 mL serum vials with a headspace of ambient air with CH_4 added to a concentration of 20 %. All incubations and experiments we performed in the dark. Liquid cultures were initially prepared from a frozen 30 % glycerol stock. Prior to distribution of inoculum to the respective acclimation temperatures, the cultures were maintained at room temperature (~20 °C) or 15 °C (*Methylobacter* sp. G7) for a period of at least six weeks, during which time multiple culture transfers ensured glycerol removal through serial dilution. The isolation of *Methylobacter* sp. G7 was initiated by using biofilm material from coal pieces as inoculum in bottles with NMS medium and 20 % CH_4 in the headspace. The enrichment culture was streaked on filters floating on NMS medium and cultivated under a headspace atmosphere with 20 % CH_4 for colony picking and isolation. The strain is currently being prepared for deposition to strain collections, and at the same time, data for a species description is being prepared.

Acclimation. During acclimation, cells were incubated in NMS medium under ~80 % air and ~20 % CH_4 (100 % CH_4 was injected into the air-containing bottle headspace) at 8, 15, 21, and 27 °C (*M. tundripaludum* SV96^T and *M. luteus* ACM 3304^T), or 4, 8, 15 and 21 °C (*Methylobacter* sp. G7) (Fig. S2). The volumes required to obtain these concentrations, resulted in ~1.3 atm headspace pressure at room temperature inside the incubation bottles. The bottles were sealed with butyl rubber plugs (3 mm thick, Chromacol, Munich, Germany) and aluminium crimp caps (Chromacol, Munich, Germany). During acclimation, the cultures were transferred to new NMS medium three times to maintain the cells in exponential growth and finally obtain cultures in early exponential phase at a density of ~1 ×

10^8 cells mL^{-1}. These cultures were diluted to approximately 5 × 10^7 cells mL^{-1} with NMS medium for growth kinetics and CH_4 oxidation kinetics experiments (Fig S2). Cells were diluted to ~1 – 3 × 10^7 cells mL^{-1} for other experiments or as a starting point for incubations to generate biomass: growth test-experiment, transcriptomics, glycogen measurements, and nucleic acids extractions. The cultures were always allowed acclimation for the time needed to surpass 10 generations (7 – 12 days). During acclimation and experiments, cultures were shaken in a horizontal position, at 50 rpm. Some control experiments were carried out at 150 rpm to test the effect on mass transfer limitations.

Shaking speed and mass transfer limitations. Strains that consume gases can experience mass transfer limitations due to low substrate solubility if, for example, high cell densities result in CH_4 uptake rates that are higher than the CH_4 dissolution rates. Gas solubility decreases with increasing temperature. Therefore, estimates of activity must be related to the dissolved concentration, and mass transfer limitation must be prevented. Faster shaking or increased headspace gas concentrations increase the rate of gas dissolution, but the former is more commonly used to prevent mass transfer limitation. We applied 150 and 50 rpm shaking (KS501, Ika Labortechnik, Staufen, Germany), for comparison of shaking speed effects (Fig. S2). At 50 rpm, the CH_4 uptake per 10^8 *M. tundripaludum* SV96^T cells was linear at all temperatures and CH_4 concentrations (SD B: Table B2). For the different temperatures, 8, 15, 21, and 27 °C, averages of R^2 (21 linear regressions per temperature in triplicates for each of seven concentrations) were 0.91, 0.91, 0.98, and 0.93, respectively (SD B: Table B2). At the two lowest concentrations, where mass transfer limitations are most probable, R^2 was above 0.9 for all temperatures (SD B: Table B2). This confirms zero order kinetics at all temperatures and CH_4 concentrations, despite increasing cell density with time (SD B: Table B1), meaning that cells growing at 50 rpm are not exposed to mass transfer limitations of any substrate needed for CH_4 oxidation, including CH_4 and O_2. To check whether mass transfer limitations occur after prolonged incubations at high CH_4 concentrations, which is relevant for evaluating potential limitations during the longer lasting growth period to accumulate enough biomass for the transcriptomics experiments, we extended some of the CH_4 oxidation experiments with *M. tundripaludum* SV96^T at 8 and 21 °C, to 22 hours. We could

show that after 22 hours, the per cell CH_4 oxidation rate is still linear (n = 9 incubations across 6 timepoints and average R^2 = 0.95 at 21 °C and 8 °C), confirming absence of mass transfer limitations for CH_4 oxidation within the timeframes relevant for our experiments (SD B: Table B15). We also tested the effect of higher shaking speed on *M. tundripaludum* SV96^T at 15 and 21 °C. When shaking at 150 rpm at CH_4 saturation, growth rates were ~0.035 – 0.073 cell divisions per cell per hour at 15 and 21 °C (Fig. S7B, C, D, and SD B: Table B16 – B18). In comparison, when shaking at 50 rpm at the same temperatures, growth rates were 0.085 – 0.11 cell divisions per cell per hour at CH_4 saturation (Fig. S4A, S7A, and SD B: Table B1, B11). This demonstrates slower average growth at 150 rpm than at 50 rpm, suggesting that increased speed might negatively affect growth due to mechanical stress.

Cell growth experiments. From samples of acclimated exponential phase *M. tundripaludum* SV96^T cultures (75ml) with densities of ~1 – 3 × 10^7 cells mL^{-1} 22 mL were transferred to three 125 mL glass bottles per temperature. Bottles were then prepared with headspaces of 80 % air and 20 % CH_4 and a pressure of 1.3 atm. This resulted in 0.36 – 0.51 mM dissolved CH_4, depending on the temperature, above the threshold of CH_4 saturation for *M. tundripaludum* SV96^T at this density (0.1 mM CH_4; Fig. S4B). Bottles were sealed with butyl rubber plugs (3 mm thick, Chromacol, Munich, Germany) and aluminium crimp caps (Chromacol, Munich, Germany). The bottles were incubated at 8, 15, 21, and 27 °C and 50 rpm (Fig. S2A) or 150 rpm (Fig. S2E) for up to 36 hours. See SD B: Table B11, B16 for incubation times and sampling time points for each experiment. At time zero and respective sampling intervals, 300 µL of cell suspension (in duplicate) was transferred to a Nunclon Delta Surface plate (Thermo Scientific, Waltham, MA, USA) and the optical density was measured (Spectra Max 250 microplate reader, Molecular Devices, San José, CA, USA) at 600 nm (diluted NMS medium as blank). For optical density measurements (OD_{600}), blanks were subtracted from measurements.

CH_4 oxidation kinetics and growth kinetics. Subsamples of acclimated exponential-phase cultures with densities of 5 × 10^7 cells mL^{-1} were aliquoted (21.6 mL) in 125 mL glass bottles for measurement of CH_4 oxidation and growth rates at seven different CH_4 concentrations and four different temperatures (8,

15, 21, and 27 °C for *M. luteus* ACM 3304^T and *M. tundripaludum* SV96^T, and 4, 8, 15, and 21 °C for *Methylobacter* sp. G7). We used three aliquots for each of the seven CH_4 concentrations, for a total of 21 bottles per temperature. With four temperatures, that amounted to a total of 84 bottles per strain (Fig. S2A, B, C, F, and G). For each temperature, two negative controls (medium without cells) in 125 mL glass bottles were prepared per CH_4 concentration (56 negative controls in total per strain). None of the negative controls indicated non-biological CH_4 consumption or leakage. To create the seven different CH_4 concentrations, seven volumes (200 µL; 600 µL; 1,5 mL; 3 mL; 6 mL; 12 mL; 15 mL) of 100 % CH_4 were injected with a plastic syringe (BD Plastipak, Franklin Lakes, NJ, USA) with a sterile 0.5 × 16 mm needle (BD Microlance, Franklin Lakes, NJ, USA) from a multi-layer polypropylene gas bag (RESTEK, Bad Homburd vor der Höhe, Germany). Final headspace concentrations of CH_4 ranged between 1000 and 130 000 p.p.m.v. while dissolved concentrations of CH_4 ranged between 1.5 and 260 µM depending also on temperature. The gas pressures in the bottles were then adjusted to a total headspace pressure of ~1.3 atm at 20 °C by injecting additional volumes of air. When adding CH_4, bottle pressure increases, resulting in lower than atmospheric concentrations of other atmospheric gases. However, partial pressures stay approximately the same. The experiments lasted up to 9 hours, during which gas samples were collected at 4 – 5 time points, including the experiment start (t0). See SD B: Tables B1 – B3, B5 – B10, B17, and B18 for the length of respective experiments, exact time points, and number of samples collected for each individual experiment. For CH_4 measurements, 0.5 mL of headspace gas was sampled with a pressure-lock 1 mL gas syringe (VICI Precision sampling, Baton Rouge, LA, USA) with a side-port needle (0.020” × 0.012” × 2”; VICI Precision sampling, Baton Rouge, LA, USA). The pressure in the syringe was adjusted to ambient pressure, before injection on a gas chromatograph (SRI 8610C, fitted with an 8600-PKDC 3 cm 9’Haysep D Column and a flame ionization detector, SRI Instruments, Torrence, CA, USA). The pressure in the bottles and the ambient pressures were monitored using a manometer (LEO 1, Keller, AG, Winterthur, Switzerland). Calibration gases were injected at the same temperature and pressure (ambient) as the samples, allowing calculation of gas concentrations and using the bottles pressures and the mass of the gases in the injected sample.

Calibration was performed using certified standards (Linde gas, Dublin, IE, and Messer AG, Bad Soden am Taunus, Germany). From the second measurement and onward (t0 was the culture batch aliquoted to serum flasks), 340 µL of the liquid culture was sampled in parallel to the headspace gas. The optical densities of these liquid samples were determined as described in the section "Cell growth experiments". These estimates of cell growth together with the OD_{600} to cell count standards described below (SD B: Table B19) were used for calculations of CH_4 oxidation per cell division and growth kinetics.

Glycogen quantification. The cells were acclimated and cultivated under 20 % CH_4 in air as described above. Five replicate bottles with 20 mL culture and 20 % CH_4 in air were incubated per temperature in darkness with 50 rpm shaking, five bottles with a starting cell concentration of ~1 × 10^7 cells mL^{-1} and five with a density of ~1 × 10^8 cells mL^{-1}. An incubation length of 48 hours at 15 and 21 °C was sufficient to reach high-density exponential growth phase at 27 °C and 8 °C it took 60 and 90 hours, respectively, to reach those same cell densities. During harvest, 2 mL of culture was sampled for glycogen measurements from the dense cultures and 10 mL from the dilute cultures. The samples were centrifuged in 2 mL Safe Lock tubes at 25 000 × g and 4 °C for 10 minutes to collect cell pellets. The cell pellets were washed in 1 mL 1 × PBS (phosphate buffered saline, pH 6.8) and stored at -80 °C. After thawing on ice, the pellets were resuspended in 400 µl ice cold milliQ water, transferred to lysing matrix E tubes (MP biomedicals, Irvine, CA, USA) for bead beating at 4.0 m s^{-1} for 15 seconds before heat treatment at 99 °C for 10 minutes and centrifugation at 10 000 × g at 4 °C for 10 min. The recovered lysate was kept on ice while preparing the glycogen kit reagents and standard dilutions for fluorometric analysis according to recommendations from the manufacturer (Glycogen assay kit, ab65620, Abcam, Cambridge, UK). Each sample was pipetted in four wells mixing 25 µL lysate with 25 µL hydrolysis buffer. Two wells of each sample and standards were added 1 µL hydrolysis enzyme each, while the remaining two wells of each sample did not receive enzyme and acted as negative controls. This was followed by incubation at 20 °C for 30 min. All wells were then added 50 µL reaction mix, incubated another 30 min at 20 °C. Glycogen was quantified using a plate reader (GloMax Explorer,

Promega, Madison, WI, USA). The glycogen was measured as oxidized glucose, originating from hydrolyzed glycogen, that was labeled with an OxiRed probe (hydrogen peroxide probe) present in the reaction mix. Emission was read at 560 nm.

OD600 to cell number conversion and cell sizes. To normalize rates to cell numbers, standard curves correlating optical density (OD_{600}) to cell numbers were created for the three strains: *M. tundripaludum* SV96^T, *M. luteus* ACM 3304^T and *Methylobacter* sp. G7. Defined volumes of cell cultures from dilution series, with known optical densities, were quantified. For each cell density, 24 × 1/25 mm^2 in two 1 mm grids of known volume were counted using a Helber cell counting chamber and light microscopy. The averages of these counts were used to calculate the number of cells per volume and correlate it to the optical densities to generate the standard curves (SD B: Table B19). For size estimation, cells were visualized using a Carl Zeiss AxioObserver Z1 with a 100x objective and Bright-Field and measured with the size estimation tool available in the AxioVision SE64 Rel 4.9.1 software. Cultures for size estimation were prepared by acclimation of cultures to 8 and 21 °C, as described above, followed by cultivation to reach exponential phase and harvest for size estimation.

Calculations. Specific growth rates were calculated as the slope of the natural logarithm of optical densities against time, during exponential growth. Mixing ratios of CH_4 and CO_2 were calculated by comparison to certified standards. Masses of headspace and dissolved CH_4 and total CO_2 at different temperatures were calculated from the mixing ratios of CH_4 and CO_2 using Henry's Law, assuming an ideal state, knowing the ambient pressure, temperature, headspace volume of the bottle, headspace pressure, liquid volume, and respective temperature-dependent solubility constants of the gases. All calculations accounted for removal of gas and liquid for measurements. We calculated the CH_4 oxidation and CO_2 production rates from slopes of linear models fitted to the concentrations measured during experiments and adjusted to the number of cells in the culture. Briefly, the oxidation between any two time points was normalized to the number of cells in the culture at that time, and as such the CH_4 or CO_2 estimated for any given time point represented what would have been measured if a

constantly sized population of cells had been active throughout the experiment. Growth efficiencies were calculated by multiplying the specific growth (cell divisions $cell^{-1}$ $hour^{-1}$) rate by 10^8 and then dividing by CH_4 the oxidation rates (µmol CH_4 oxidized 10^8 $cells^{-1}$ $hour^{-1}$) to give cell divisions per µmol CH_4 oxidized. The data used to estimate growth efficiency were the values predicted for each measurement time-point from the non-linear Michaelis-Menten regression models. For estimates of specific growth rates, CH_4 oxidation, and growth efficiency at CH_4 saturation, $V_{max(app)}$ values predicted from the Michaelis-Menten kinetics models were used (see below for $V_{max(app)}$ estimation).

Statistics for physiological data. Tests of significant differences in cellular RNA, DNA, and protein content, CO_2 production rates, growth rates, and glycogen content between cells at different temperatures were done using the built-in function for the t-test in R (5) ("pairwise.t.test"). All *p* values were corrected for multiple testing using the method of Benjamini and Hochberg (6) within the R function "p.adjust" (5) (SD B: Table B20). The Michaelis-Menten CH_4 oxidation and growth kinetics of *M. tundripaludum* SV96^T, *M. luteus* ACM 3304^T, and *Methylobacter* sp. G7 were modelled using the "nls" function of the "nlstools" R package (7), specifying the "michaelis" model and providing start values for $K_{m(app)}$ and $V_{max(app)}$ (Km=0.002, Vmax=0.02). Kinetics coefficients and corresponding *p* values were extracted from these models using the "summary" function. Modelled growth and CH_4 oxidation rates were predicted using the "predict" function and used to estimate cell divisions per µmol CH_4 oxidized at different dissolved CH_4 concentrations. Four-parameter logistics curves to model the growth efficiency (cell divisions per µmol CH_4 oxidized) at different dissolved CH_4 concentrations were fitted using the "drm" function of the "drc" package (8). Functions of the "R.utils" and "plyr" packages were used in addition to built-in functions to handle the data in R (9), while "ggplot2" (10), in addition to built-in functions, was used for linear modelling (function "*lm"*) and analysis, and to plot the data.

RNA and DNA extraction, and sequencing. Six cultures of *M. tundripaludum* SV96^T were acclimated at each of the temperatures, 8, 15, 21, or 27 °C, as described above. After acclimation, the cultures were cultivated to a sufficient cell density for extraction in exponential phase growth. Cultures at 8 and 27

°C were incubated for 36 hours, and cultures at 15 and 21 °C for 16 hours, prior to harvest for RNA and DNA extractions. This allowed the cells to reach sufficient density for the downstream approaches. The cultures were pelleted by centrifugation and re-suspended in 740 µL TE buffer (pH 8). Further, 40 µL lysozyme (50 mg/mL) was added, and the suspensions were incubated for 5 min at room temperature. Then, 40 µL of 10 % SDS and 8 µL of proteinase K (10 mg/mL) were added, and the samples were incubated at 37 °C for 1 h. Next, 100 µL of 5 M NaCl and 100 µL cetyl trimethylammonium bromide (50 g/L) /NaCl (20 g/L) were added, and the samples were incubated at 65 °C for 10 min. Nucleic acids were then extracted with one volume phenol/chloroform/isoamyl alcohol, followed by extraction with one volume of chloroform/isoamyl alcohol (25:24:1) and finally precipitated with 0.6 volumes of isopropanol and dissolved in RNase free water. From these samples, DNA was removed using the TURBO DNA-free kit (Ambion, Thermo Fisher Scientific, Waltham, MA, USA) prior to sequencing. DNA and RNA were quantified using Qubit (Thermo Fisher scientific, Waltham, MA, USA), prior to DNA removal. RNA quality was assessed using automated gel electrophoresis with Experion (Bio-Rad, Hercules, CA, USA), demonstrating high quality RNA with 23S to 16S rRNA ratios of 1.6 – 2.4. The quantity of RNA and DNA per 10^8 cells was calculated by dividing the mass of extracted RNA by the number of cells used for the extraction. Three extracts per temperature, in which DNA had been digested (see above), were processed further. Ribosomal RNA was removed from the total RNA of these 12 samples using the Ribo-Zero rRNA Removal Kit (Bacteria, Epicentre, Illumina, San Diego, CA, USA), and the remaining RNA was sequenced. Transcriptomic libraries were prepared from the rRNA depleted samples using the Illumina TruSeq RNA-seq library preparation kit v1, following the manufacturers recommendations and sequenced using 2 × 100 bp paired-end technology on the HiSeq 2000 platform (Illumina). This procedure allowed the generation of high-coverage transcriptomes for analysis of gene transcription.

Computational analyses. Sequences were trimmed with Trimmomatic, using default settings (11), to remove Illumina adapters (ILLUMINACLIP:ADAPTERS:2:30:10), removing bases from the start and end of the sequence if below a quality threshold of 3 (LEADING:3, TRAILING:3), and remove four-base-pair

sequence sections that fell below a quality threshold of 15 (SLIDINGWINDOW:4:15), as well as any sequences shorter than 36 bp (MINLEN:36). The rRNA sequences were removed using SortMeRNA (12), with default settings and the 5S, 16S and 23S rRNA gene copies from the *M. tundripaludum* SV96^T genome as reference sequences. Non-ribosomal RNA was mapped to the *M. tundripaludum* SV96^T genome using BWA *sampe* (to generate alignments in the SAM format given paired-end reads aligner) (13) with default settings. The number of different genome features present in the transcriptomes were counted using FeatureCounts (14). Features were counted if both paired reads were assigned to the same feature, and these were then counted as one fragment. Gene expression data was analysed using R (5). To compare expression between samples, matrices holding the counts of genome features in each of the 12 transcriptomes were normalized using the median of ratios method available in the R package DEseq2 (15). This method uses the median of ratios of transcript counts for different genes in a sample to their respective geometric means (the mean of transcript counts for a particular gene across all samples). To test whether differential expression between different temperatures were significant, we applied the Wald test with multiple testing correction using the false discovery rate (FDR) controlling method of Benjamini and Hochberg (6), the default statistical test for differential expression in DEseq2. This method is highly robust with three or more replicates, producing very few false positives (< 5 %) regardless of fold-change thresholds (16), but it is prone to false negatives at low replication numbers (≤ 6). This means that the risk that significant biological patterns across multiple genes are false positives, is negligible with three replicates. However, there is significant risk of failing to identify changes that occur.

Gene expression data were visualized using the R package *Heatmap3*. Based on the genome of *M. tundripaludum* SV96^T (17), we reconstructed central carbon and energy metabolisms, pathways of protein biosynthesis and functions relevant to cell division and other cell structures by manually curating the annotations for the genome of *M. tundripaludum* SV96^T available in Genoscope (https://mage.genoscope.cns.fr/microscope/mage/viewer.php?O_id=818). The *M. tundripaludum* SV96^T genome is also available from NCBI (90) with accession number “NZ_AEGW00000000.2”.

Maps. To assess the global distribution of the genus *Methylobacter* we screened the publicly available 16S rRNA gene fragment collection of the Earth Microbiome Project (EMP), a large-scale collaborative project employing standardized collection, curation, and analysis of environmental samples (18-20). Sample processing, sequencing, and core amplicon data analysis were performed by the Earth Microbiome Project (www.earthmicrobiome.org), and all amplicon sequence data and metadata have been made public through the EMP data portal (qiita.microbio.me/emp). To obtain putative *Methylobacter* 16S rRNA gene fragments we selected all EMP 16S rRNA gene fragments with ≥99 % sequence identity to the 16S rRNA genes of *M. tundripaludum* SV96^T, *M. luteus* ACM 3304^T, *Methylobacter* sp. G7, *Methylobacter whittenburyi* ACM 3310, *Methylobacter marinus* A45, and *Methylobacter psychrophilus* Z-0021 identified via BLASTn search (21). A total of 59 putative *Methylobacter* 16S rRNA gene fragments were retrieved from the three EMP datasets (19) (emp_deblur_90bp.qc_filtered.biom, emp_deblur_100bp.qc_filtered, emp_deblur_150bp.qc_filtered.biom), which comprise three different sequence lengths, 90 bp, 100 pb, and 150 bp, respectively. The retrieved 59 putative *Methylobacter* 16S rRNA gene fragments were further evaluated by checking whether any of the fragments had a higher identity to 16S rRNA genes from non-*Methylobacter* closely related to *Methylobacter.* The selection of non-*Methylobacter* strains were based on a phylogenomic analysis of the order *Methylococcales* (22) and included *Methylosarcina lacus* LW14, *Methylovolum psychrotolerans* Sph1, *Methylocucumis oryzae* Sn10-6, *Chlonothrix fusca* AW-b, *Methylosoma difficilie* LC 2, *Methyloprofondus sediimenti* WF1, *Methylosphaera hansonii* AM6, *Mehylovulum miyakonense* HT12, *Methyloglobus morosus* KoM1, *Methylomicrobium agile* ATTC35068, *Methylomicrobium album* BG8, *Methylomicrobium pelagicum* AA-23, *Methylomicrobium japanense* NI, *Methylomicrobium alcaliphilum* 20Z, *Methylomicrobium buryatense* 5B, *Methylomicrobium kenyense* AMO1, *Methylomarinum vadi* IT-4*, Methylomonas lenta* R-45377, *Methylomonas rubra* 15sh, *Methylomonas scandinavica* SR5, *Methylomonas paludis* MG30*, Methylomonas denitrificans* FJG1, *Methylomonas methanica* NCIMB11130, *Methylomonas koyamae* JCM16701, *Methylomonas fodinarum* LD2, *Methylomonas aurantiaca* JB103*, Methylosarcina*

quisquiliarum AML-D4*, Methylosarcina fibrata* AML-C10*, Methylococcus mobilis* LMD 77.28. In total, 12 of the 16S rRNA gene fragments showed ≥99% sequence identity to close related taxa and were thus excluded. The 23,813 available EMP samples (all released EMP samples that included coordinates), representing 1,294 unique geographic locations, were screened for the occurrence and relative abundance of the remaining 42 *Methylobacter* 16S rRNA gene fragments. Due to the overlap between the three screened EMP datasets, duplicated samples were removed, keeping the entry with the highest relative abundance of *Methylobacter*. R (5) (version 4.1.3) was used to analyse environmental distribution and relative abundances of *Methylobacter* 16S rRNA gene fragments and graphically display the results (Rstudio version 2022.02.2+485) including the R packages "tidyverse" version 1.3.1 (23), "maps" version 3.4.0 (https://cran.r-project.org/web/packages/maps/), and "mapdata" version 2.3.0 (https://cran.r-project.org/web/packages/mapdata/). Adobe Illustrator (CC 26.3.1.) was used for final figure editing.

Data deposition and scripts for computational analyses. The RNA-seq data from this study have been deposited under the accession project number PRJNA390985 in the NCBI short read archive (SRA). The individual experiment identification numbers for each of the 12 datasets are found in SD B: Table B13. Scripts for RNA-seq pre-processing, gene expression analysis, statistical tests, modelling, and visualization in R, are provided online (ALEXANDER Tveit, 2022, "Thermal acclimation of methanotrophs from the genus Methylobacter", https://doi.org/10.18710/Z1QF8W, DataverseNO). Data for maps are provided in SD A. Physiological data are provided in SD B. The processed and normalized transcript counts and statistics for the differential gene expression analyses are provided in SD C.

References

1. Wartiainen I, Hestnes AG, McDonald IR, Svenning MM (2006). *Methylobacter tundripaludum* sp. nov., a methane-oxidizing bacterium from Arctic wetland soil on the Svalbard islands, Norway (78 degrees N). *Int J Syst Evol Microbiol* 56: 109 – 113.

2. Bowman JP, Sly LI, Nichols PD, Hayward AC (1993). Revised Taxonomy of the Methanotrophs: Description of *Methylobacter* gen. nov., Emendation of *Methylococcus*, Validation of *Methylosinus* and *Methylocystis* Species, and a Proposal that the Family *Methylococcaceae* Includes Only the Group I Methanotrophs. *International Journal of Systematic and Evolutionary Microbiology* 43: 735 – 753.

3. Whittenbury R, Phillips KC, Wilkinson JF (1970). Enrichment, isolation and some properties of methane-utilizing bacteria. *Journal of general microbiology* 61: 205 – 218.

4. Pfennig N (1962). Beobachtungen über das Schwärmen von *Chromatium okenii*. *Archiv für Mikrobiologie* 42: 90 – 95.

5. R_Development_Core_Team (2009). R: A language and environment for statistical computing. R Foundation for Statistical Computing: Vienna, Austria.

6. Benjamini Y, Hochberg Y (1995). Controlling the False Discovery Rate: A Practical and Powerful Approach to Multiple Testing. *Journal of the Royal Statistical Society Series B (Methodological)* 57: 289 – 300.

7. Baty F, Ritz C, Charles S, Brutsche M, Flandrois J-P, Delignette-Muller M-L (2015). A Toolbox for Nonlinear Regression in R: The Package nlstools. *Journal of Statistical Software* 66: 1 – 21.

8. Ritz C, Baty F, Streibig JC, Gerhard D (2016). Dose-Response Analysis Using R. *PLOS ONE* 10: e0146021.

9. Wickham H (2011). The Split-Apply-Combine Strategy for Data Analysis. *Journal of Statistical Software* 40: 1 – 29.

10. Wickham H (2009). *ggplot2: Elegant Graphics for Data Analysis*. Springer-Verlag New York.

11. Bolger AM, Lohse M, Usadel B (2014). Trimmomatic: a flexible trimmer for Illumina sequence data. *Bioinformatics* 30: 2114 – 2120.

12. Kopylova E, Noe L, Touzet H (2012). SortMeRNA: fast and accurate filtering of ribosomal RNAs in metatranscriptomic data. *Bioinformatics* 28: 3211 – 3217.

13. Li H, Durbin R (2009). Fast and accurate short read alignment with Burrows-Wheeler transform. *Bioinformatics* 25: 1754 – 1760.

14. Liao Y, Smyth GK, Shi W (2014). featureCounts: an efficient general purpose program for assigning sequence reads to genomic features. *Bioinformatics* 30: 923 – 930.

15. Anders S, Huber W (2010). Differential expression analysis for sequence count data. *Genome Biol* 11: R106.

16. Schurch NJ, Schofield P, Gierliński M, Cole C, Sherstnev A, Singh V *et al* (2016). How many biological replicates are needed in an RNA-seq experiment and which differential expression tool should you use? *Rna* 22: 839 – 851.

17. Svenning MM, Hestnes AG, Wartiainen I, Stein LY, Klotz MG, Kalyuzhnaya MG *et al* (2011). Genome Sequence of the Arctic Methanotroph Methylobacter tundripaludum SV96. *J Bacteriol* 193: 6418 – 6419.

18. Gilbert JA, Meyer F, Antonopoulos D, Balaji P, Brown CT, Brown CT *et al* (2010). Meeting report: the terabase metagenomics workshop and the vision of an Earth microbiome project. *Stand Genomic Sci* 3: 243 – 248.

19. Thompson LR, Sanders JG, McDonald D, Amir A, Ladau J, Locey KJ *et al* (2017). A communal catalogue reveals Earth's multiscale microbial diversity. *Nature* 551: 457 – 463.

20. Gilbert JA, Jansson JK, Knight R (2014). The Earth Microbiome project: successes and aspirations. *BMC Biology* 12: 69.

21. Camacho C, Coulouris G, Avagyan V, Ma N, Papadopoulos J, Bealer K *et al* (2009). BLAST+: architecture and applications. *BMC Bioinformatics* 10: 421.

22. Orata FD, Meier-Kolthoff JP, Sauvageau D, Stein LY (2018). Phylogenomic Analysis of the Gammaproteobacterial Methanotrophs (Order Methylococcales) Calls for the Reclassification of Members at the Genus and Species Levels. *Frontiers in Microbiology* 9: 3162.

23. Wickham H AM, Bryan J, Chang W, McGowan LD, François R, Grolemund G, Hayes A, Henry L, Hester J, Kuhn M, Pedersen TL, Miller E, Bache SM, Müller K, Ooms J, Robinson D, Seidel DP, Spinu V, Takahashi K, Vaughan D, Wilke C, Woo K, Yutani H (2019). Welcome to the tidyverse. *Journal of Open Source Software* 4: 1686.

24. Kimura M (1980). A simple method for estimating evolutionary rates of base substitutions through comparative studies of nucleotide sequences. *J Mol Evol* 16: 111 – 120.

25. Kumar S, Stecher G, Li M, Knyaz C, Tamura K (2018). MEGA X: Molecular Evolutionary Genetics Analysis across Computing Platforms. *Mol Biol Evol* 35: 1547 – 1549.

26. Söllinger A, Séneca J, Dahl MB, Motleleng LL, Prommer J, Verbruggen E *et al* (2022). Down-regulation of the bacterial protein biosynthesis machinery in response to weeks, years, and decades of soil warming. *Science Advances* 8: eabm3230.
